# The kaolinite shuttle links the Great Oxidation and Lomagundi events

**DOI:** 10.1038/s41467-021-23304-8

**Published:** 2021-05-19

**Authors:** Weiduo Hao, Kaarel Mänd, Yuhao Li, Daniel S. Alessi, Peeter Somelar, Mathieu Moussavou, Alexander E. Romashkin, Aivo Lepland, Kalle Kirsimäe, Noah J. Planavsky, Kurt O. Konhauser

**Affiliations:** 1 Department of Earth and Atmospheric Sciences, University of Alberta, Edmonton, Canada; 2 Department of Geology, University of Tartu, Tartu, Estonia; 3 Department of Geology, University of Science and Technology of Masuku, Franceville, Gabon; 4 Institute of Geology, Karelian Science Centre, Petrozavodsk, Russia; 5 CAGE—Centre for Arctic Gas Hydrate, Environment and Climate, Department of Geosciences, UiT The Arctic University of Norway, Tromsø, Norway; 6 Geological Survey of Norway (NGU), Trondheim, Norway; 7 The Department of Earth and Planetary Sciences, Yale University, New Haven, CT USA

**Keywords:** Element cycles, Geochemistry, Geochemistry

## Abstract

The ~2.22–2.06 Ga Lomagundi Event was the longest positive carbon isotope excursion in Earth’s history and is commonly interpreted to reflect perturbations in continental weathering and the phosphorous cycle. Previous models have focused on mechanisms of increasing phosphorous solubilization during weathering without focusing on transport to the oceans and its dispersion in seawater. Building from new experimental results, here we report kaolinite readily absorbs phosphorous under acidic freshwater conditions, but quantitatively releases phosphorous under seawater conditions where it becomes bioavailable to phytoplankton. The strong likelihood of high weathering intensities and associated high kaolinite content in post-Great-Oxidation-Event paleosols suggests there would have been enhanced phosphorus shuttling from the continents into marine environments. A kaolinite phosphorous shuttle introduces the potential for nonlinearity in the fluxes of phosphorous to the oceans with increases in chemical weathering intensity.

## Abstract (web summary)

Expanded phosphorus availability possibly triggered a marine bioproduction boom after 2.3 billion years ago, but its delivery mechanisms remain unclear. Here we propose a kaolinite shuttle which efficiently adsorbs phosphorus in continental weathering settings and releases it under marine conditions.

## Introduction

The LE is the most pronounced and long-lived positive carbon isotope excursion in Earth history. Marine carbonate rocks deposited during the LE, which lasted at least 160 million years, are characterized by *δ*^13^C_carb_ values >10 (refs. ^1–4^). The most widely accepted explanation for the LE relies on significant perturbation in the balance of different C burial fluxes—since organic carbon (C_org_) is depleted in ^13^C, an increase in C_org_ burial compared to carbonate burial will result in ^13^C-enrichment of the dissolved marine carbonate pool^2^. Although it is likely that at least some of the carbonate *δ*^13^C values reflect local, instead of global, processes (e.g., ref. ^5^), current explanations for the anomalously positive values require high rates of marine primary productivity. There is increasing consensus that P is likely to have been the ultimate limiting nutrient through Earth’s history^6,7^. Accordingly, explanations for the LE must center around the factors which can induce >100 myr perturbations to the global P cycle.

Oxidation of a reduced mineral enriched upper continental crust during the LE provides one possible mechanism to drive extended changes in the phosphorous cycle^8,9^. The proliferation of aerobic chemolithoautotrophy would have facilitated the rapid biological weathering of crustal sulfide minerals, leading to an extended episode of acid rock drainage, and solubilization of nutrient-bearing minerals^10^ (e.g., apatite in rocks and soils, the weathering of which is highly pH-dependent^11^). Similarly, extensive oxidation of siderite in aerially exposed sediments (e.g., shales, marls) during the LE would have led to high acid fluxes into soils and potentially promoted high atmospheric CO_2_ levels, increasing weathering regimes more broadly^9^. Elevated siderite and pyrite contents in the weatherable shell during the LE were simply a consequence of higher fluxes of reduced S and Fe to marine sediments during the Archean (e.g., refs. ^12–14^). Upon weathering, P appears to have been mostly leached away and carried into the oceans, instead of retained in the paleosol (for instance, Hao et al.^15^ report a peak in paleosol P loss at ~2.3–2.4 Ga). Kinetic and thermodynamic models further indicate that the weathering flux of P increased through the Archean, possibly reaching modern levels by the end of the eon^15^.

This driver of the LE assumes that higher acid fluxes will lead to greater P fluxes to the biosphere^8,10^. However, in the modern, typically <10% of total P is transported via rivers as dissolved anions (for detailed discussion see supplementary note 1). Most is transported in the particulate phase^16^, including P adsorbed onto Fe(III)-Al(III)-oxyhydroxides or clay minerals, or organically complexed^17^ (see Table S1). A large portion of adsorbed P is then sedimented in estuaries and marginal marine environments with the flocculation of particulate phases where they remain inaccessible to plankton until remobilization within the sediment pile^18^. In the absence of plants, the only organic compounds delivered to the oceans would have been from degraded continental microbial mats^19^, which are unlikely to have had as large of an effect on the P cycle. Further, during the LE, ferric oxyhydroxides may have been solubilized in the generally sulfide and siderite-rich Paleoproterozoic soils, in a process similar to what is observed in some pyrite-rich black shale weathering environments today^20^. Building from this framework, it seems reasonable that clay minerals were an important vector by which P was delivered to the oceans at that time.

Different terrestrial weathering regimes will lead to the formation of widely varying secondary mineral assemblages—potentially leading to major changes in P sorption and transport to the oceans. In highly weathered soils today, kaolinite [Al_2_Si_2_O_5_(OH)_4_)], and its hydrated form halloysite [Al_2_Si_2_O_5_(OH)_4_·2H_2_O], are often the only remaining clay mineral phases aside from Fe(III)- and Al(III)-oxyhydroxides and quartz^21^. With higher weathering intensities due to a stronger acid flux from pyrite and siderite oxidation, as well as higher CO_2_ levels, kaolinite formation would have been widespread. This link is well developed in modern settings, with extensive kaolinite formation in soils developed on organic and pyrite-rich lithologies such as black shales^22,23^. Despite still constituting an important part of the clay fraction, clay minerals more characteristic of moderate terrestrial weathering conditions (e.g., smectites) are more rapidly dissolved in acidic environments^21,24–26^.

Conceptually, increased fluxes of kaolinite generation post-GOE are also expected due to an increase in continental area at 2.4 Ga^27–29^, and intensified continental weathering in the warm climate aftermath of Huronian global glaciation^30–32^. While goethite and hematite formation under an oxygenated atmosphere may have driven increased Al substitution into these phases at the expense of kaolinite formation^33,34^, we consider it unlikely to have substantially decreased kaolinite fluxes, as ferric hydroxide solubility increases dramatically with lower pH and Fe^3+^ becomes the dominant pyrite oxidant in high acid production drainage environments^9^. Thus, in this work, we show the evidence of kaolinite generation in shales and paleosols post-GOE, and the capacity of kaolinite shuttling P from land to the oceans. The subsequent bacteria growth experiments demonstrate that the delivery of P by kaolinite leads to the augmentation of coastal primary productivity.

## Results

### Kaolinite in post-GOE shales and paleosols

**Fig. 1:**
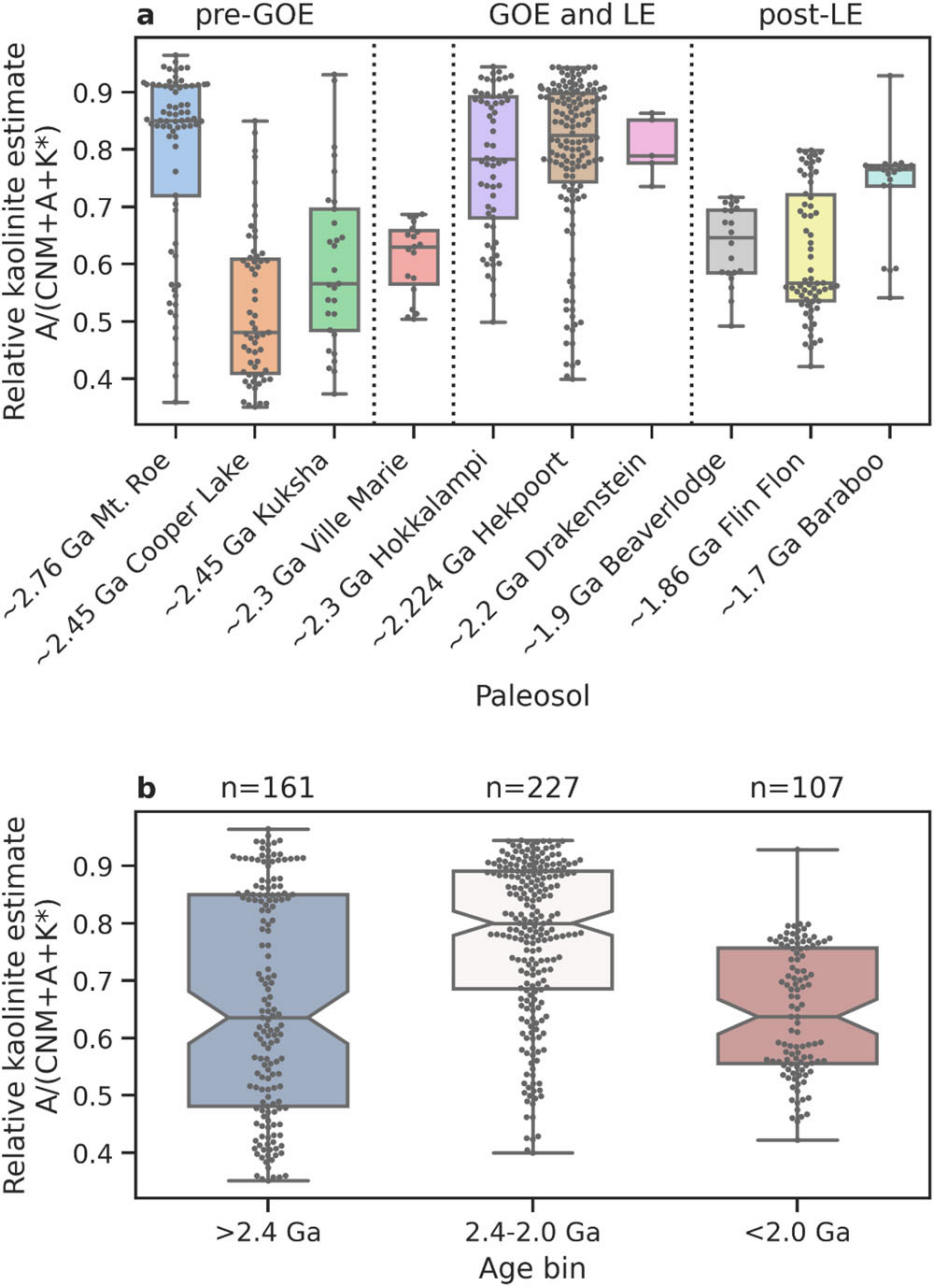
Major element composition of Late Archean to Paleoproterozoic paleosols. Relative kaolinite contents are approximated by molar Al-richness, as compared to Ca, Na, Mg, and K (CNM = CaO* + Na_2_O + MgO*; A = Al_2_O_3_; K* = K_2_O*; asterisks denote data corrected for non-silicate contribution and K-metasomatism; Methods). **a** Al-richness in individual paleosols. The Ville Marie paleosol is poorly dated and may have formed either prior to or after the Great Oxidation Event (GOE), while its profile is possibly truncated with only the saprolite part preserved. Boxes show the mean value (horizontal line) and 25%/75% quantiles (edges), whiskers show the full range. Superimposed dots denote individual samples. **b** Al-richness in paleosols within the pre-GOE (>2.4 Ga), GOE and LJE (2.4–2.0 Ga), and post-LJE (>2.4 Ga) age bins.

Consistent with the above view that an increase in kaolinite formation in the aftermath of the GOE, kaolinite is found in several LE successions. For example, the LST-12 drill core exposing the upper strata of the Francevillian Group in Gabon hosts organic-rich shales with sedimentary kaolinite alongside mixed-layer illite-smectite^35^ (supplementary note 2, and Figs. S1–S3). Despite the greenschist facies metamorphic overprint^36^, tentative traces of preserved kaolinite are also found in X-ray diffraction spectra in two shale intervals of drill core OPH, which intersects the Zaonega Formation in Russia (supplementary note 2 and Fig. S1). There is also enhanced formation of kaolinite in paleosols formed around, and following, the GOE indicating intensive acidic continental weathering at that time^37–46^. However, it is important to note that it is not possible to estimate exactly how common kaolinite is from the rock record given that it is often transformed during burial metamorphism^47^. Nonetheless, clues about the extent of kaolinite formation and weathering intensity can be gleamed from the major elemental composition of paleosols. We examine Al-richness (compared to Ca, Na, Mg, and K) in a record of definitive, relatively well-preserved paleosols through the late Archean and early Proterozoic (*n* = 10; see supplementary note 3 and Supplementary Data 1), since kaolinite is the main Al-bearing phase in highly weathered soils. Some Archean paleosols, such as Mt. Roe^45^ (Fig. 1a), display strong Al enrichment, suggesting strong weathering and local kaolinite production either under a high-CO_2_ atmosphere during long period of weathering^39^, or in the presence of transient oxygenated conditions^45^. Notably, we observe that Al-richness peaks in soils formed between 2.4 Ga and 2.0 Ga (GOE and LE), compared to paleosols older or younger than this time bracket (Fig. 1a, b), suggesting that kaolinite was highly abundant in contemporary terrestrial weathering environments. This trend is not likely an artifact of the differing latitude at which these paleosols formed given that calculated paleolatitudes do not correspond to the above trend (Supplementary Data 2).

### The shuttling of P by kaolinite from rivers to the oceans

**Fig. 2:**
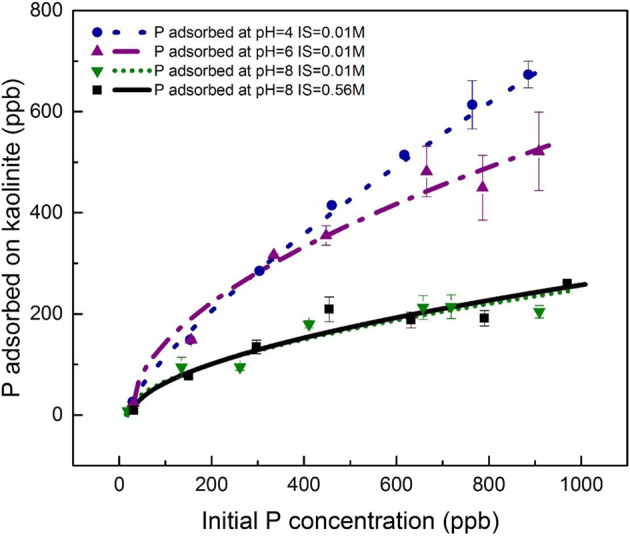
The adsorption of P onto kaolinite at four different environmental conditions. Black squares and continuous line represent P adsorption at pH 8, IS = 0.56 M conditions; green downward-facing triangles and dotted line represent P adsorption at pH 8, IS = 0.01 M conditions; purple upward-facing triangles and dashed-dotted line show P adsorption at pH 6, IS = 0.01 M conditions; blue circles and dashed line are P adsorption at pH 4, IS = 0.01 M conditions. Error bars represent the ±1 standard deviation of replicates.

We used static and dynamic adsorption experiments to demonstrate that kaolinite will more effectively shuttle P to the oceans than other typical clay minerals or oxides/hydroxides. In the static experiments we simulated freshwater (pH 4 and ionic strength 0.01 M) to seawater-like (pH 8 and ionic strength 0.56 M) aqueous conditions, and also tested P adsorption to kaolinite at various initial phosphate concentrations to bracket modern riverine concentrations (Table S1). We stress that these conditions are meant to be representative of fresh and marine waters – they are not based on empirical- or model-based reconstruction of past pH values. For all initial P concentrations <400 ppb (12.9 μM), the difference between the equilibrium adsorption experiments of simulated freshwater and seawater was negligible (Fig. 2). By contrast, at initial P concentrations >400 ppb, there was ~100–200 ppb (3.2–6.4 μM) more P adsorbed in the freshwater environment than seawater. Surprisingly, ionic strength variations were found to be insignificant in modulating P adsorption compared to pH (similar trendlines for IS = 0.01 M and 0.56 M at the pH 8 conditions; Fig. 2). However, the surface charge of kaolinite is pH-dependent^48,49^. Surface complexation modeling based on acid-base titrations of kaolinite shows that kaolinite surface functional groups are highly protonated at pH < 4.7, lending the clay a net positive charge (supplementary note 4), which can electrostatically attract negatively charged P species (dominantly H_2_PO_4_^−^ at pH 2–7). A bidentate mononuclear surface complex of P onto positively charged sites is the dominant adsorption mechanism on kaolinite, explaining the pH-dependence of P adsorption (supplementary note 4).

To compare the results to clays more typical of moderate weathering regimes, we also examined the P adsorption capacities of illite and montmorillonite in both freshwater and marine conditions (data shown in supplementary note 5). We show that even under lower degrees of weathering characterized by montmorillonite and illite production, the transport of clays from rivers to the oceans can act as a P source simply due to variations in aqueous conditions. Importantly, however, the magnitude of P release is depressed when compared to kaolinite, suggesting that the kaolinite shuttle is more conducive to transporting P from land to the sea.

**Fig. 3:**
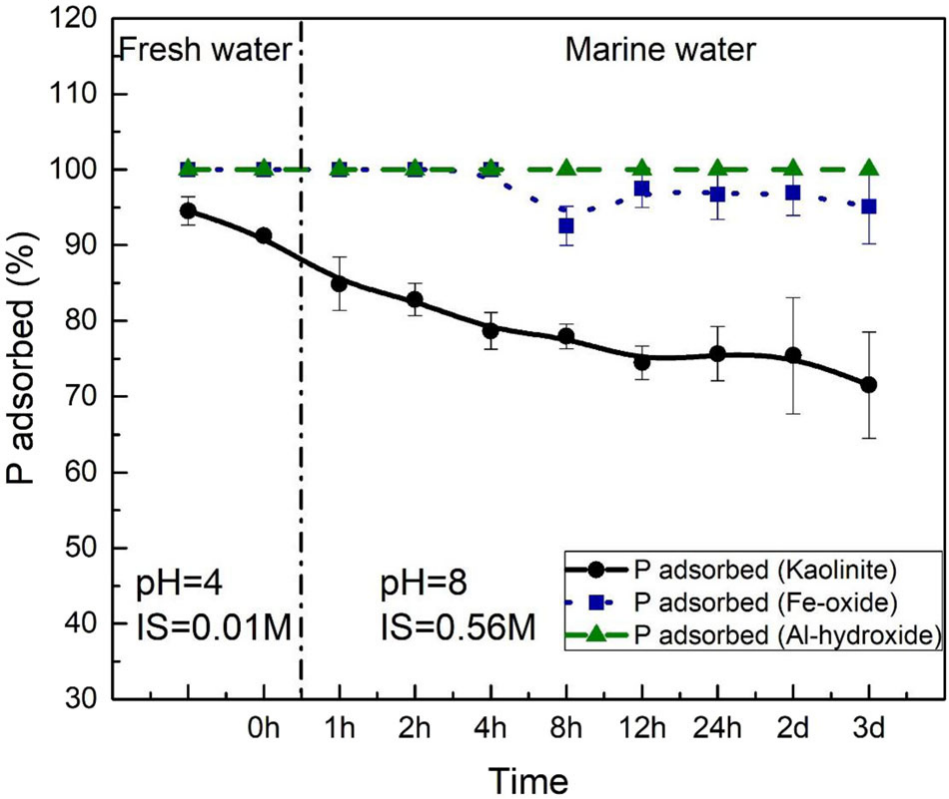
Desorption of P from kaolinite, Fe-oxide and Al-hydroxide surfaces when transitioning from freshwater to marine conditions. Freshwater conditions were simulated at pH 4 and ionic strength = 0.01 M, while marine conditions refer to pH 8 and ionic strength = 0.56 M. The black circles and continuous line represent P desorption from kaolinite surfaces; blue squares and dotted line show the P desorption from Fe-oxide; green triangles and dashed line show P desorption from Al-hydroxide. Error bars represent the ±1 standard deviation of replicates.

Dynamic adsorption experiments were performed to directly observe P behavior on kaolinite surfaces under simultaneously changing pH and ionic strength conditions (Fig. 3). At a P concentration of ~155 ppb (5 μM), 95% of the P was adsorbed onto kaolinite surfaces at shale weathering conditions (pH 4). After switching to marine conditions (pH of 8), the proportion of adsorbed P steadily declined throughout our experimental period (3 days). In total, 20% of pre-adsorbed P was released into the aqueous environment. As a comparison, we also analyzed P sorption to Fe(III)-oxides and Al(III)-hydroxide, such as hematite (Fe_2_O_3_) and gibbsite (Al[OH]_3_), respectively, as both minerals represent stable mineral oxide phases under extreme weathering environments (e.g., laterites^23,50^). For both hematite and gibbsite, we show 100% adsorption of P at freshwater conditions but negligible release at seawater pH and ionic strength (Fig. 3).

### Augmentation of nearshore primary bioproductivity

**Fig. 4:**
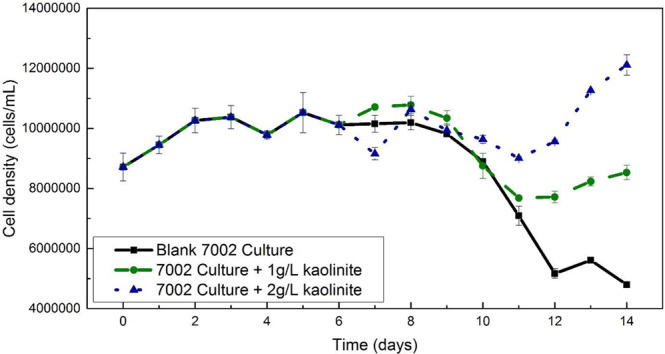
Growth curves of *Synechococcus* sp. PCC7002 at different levels of added P-bearing kaolinite. The original culture was divided into three 150 mL sub-cultures after day 6. Black squares and straight line represent the control culture incubated in modified A + growth media with only 5 µM potassium phosphate. The green circles and dashed line show the growth of a culture with addition of 150 mg of P-bearing kaolinite after day 6. The blue triangles and dotted line represent the culture in which 300 mg of P-bearing kaolinite was added after day 6. Error bars represent the ±0.5 standard deviation of replicates.

To confirm that the desorbed P in our experiments will be truly bioavailable, we also explored the impact of P bearing kaolinite input to the growth of the marine cyanobacterium *Synechococcus* sp. PCC 7002 in continuous culture; this species comprises a significant fraction of the modern marine phytoplankton^51^. Without clay input, cell density reached a plateau at the 2nd day and then decreased to 5 × 10^6^ cells/mL (55% of initial density) at the 14th day, while with 1 g/L of P-bearing clay input, the cell number reached 8 × 10^6^ cells/mL (97% of initial density) at the end of the growth period. A 2 g/L P-bearing kaolinite input led to a bacterial cell density of 1.2 × 10^7^ (139% of initial density), more than twice that of the blank (Fig. 4). In summary, the high adsorption capacity of kaolinite under freshwater conditions and its low capacity under marine conditions results in enhanced P bioavailability and cyanobacterial productivity in post-GOE nearshore environments.

## Discussion

It should still be noted that kaolinite shuttling would not have been the sole process providing bioavailable P to marine primary producers after the GOE. For example, although P release from Fe(III)- and Al(III)-hydroxides is not as efficient as from kaolinite, bound P can be released from sediments to the water column over longer periods during early diagenesis^52^. Additionally, since Paleoproterozoic oceans were likely redox-stratified, some ferric oxyhydroxides would have been reductively dissolved below the redoxcline, and the released P made bioavailable through upwelling^53^. Smectite (montmorillonite) and illite, though showing a relatively lower P shuttling capacity compared to kaolinite (supplementary note 5), may also have contributed to nutrient shuttling during the Lomagundi Event, as, again, suggested by the preservation of smectite-rich clay minerals in Francevillian black shales^35,54^ and major elemental compositions close to average smectite composition in contemporary paleosols (Fig. S3). It is also worth noting that any changes in terrestrial P inputs are going to be modulated by the marine P cycle. For instance, coupled P and Fe enrichment in the Francevillian basin, which was likely influenced by upwelling (e.g., refs. ^55,56^), is likely a signal of Fe control on marine P cycling. Nonetheless, we highlight that the kaolinite shuttle added upon, and compounded, these prior P-fertilization mechanisms to significantly increase the supply of bioavailable P to nearshore ecosystems.

It is also possible that the kaolinite shuttle was turned on during other extreme global carbon cycle and biogeochemical perturbations in Earth’s past marked by anomalously high primary productivity (e.g., the Permian Triassic biotic crisis; Paleocene-Eocene Thermal Maximum (PETM)). For example, the PETM is characterized by a rise of surface temperature (5–8 °C) and extensive continental weathering (e.g., ref. ^57^). Consistent with our view, there are spikes in kaolinite formation during the PETM in several regions^58,59^. A compilation of marine sediments from the P-E boundary demonstrates a dramatic increase in kaolinite generation (approaching 80% of kaolinite in clay phases) during the PETM period (supplementary note 6, Supplementary Data 3, and Fig. S11). The shuttle of kaolinite from land to the oceans would have increased marine bio-productivity, induced the storage of CO_2_ into ^13^C-depleted organic carbon, and subsequently recover the post-PETM *δ*^13^C_carb_ to Paleocene values (see ref. ^57^).

The kaolinite shuttle provides a novel mechanism to tie together known threads of Earth’s biogeochemical environment following the initial rise of atmospheric oxygen – increased acid weathering and enhanced primary productivity. A critical aspect of the model is that the response of the P cycle to weathering intensity is non-linear. Phosphorous fluxes became greatly enhanced when intense terrestrial weathering conditions that produced abundant kaolinite became spatially widespread. Strong nonlinearity in the extent of P shuttling could help explain why the Lomagundi stands out as a unique event in Earth’s history. Reconstructing mineralogical trends in deep time events and from Lomagundi-aged sediments is difficult given clay transformation with burial, but more recent “weathering events” (e.g., the PETM) bolster the case that there is a link between terrestrial weathering regimes, nutrient fluxes, intervals of anoxia, extinction, and ecosystem transitions.

## Methods

The paleosol chemical database of eight relatively complete paleosols ranging in age from 2.76 Ga to 1.70 Ga was compiled from published paleosol studies and used to assess trends in weathering intensity through the time period. Al, Ca, Na, Mg, and K were plotted on a CNM-A-K ternary plot, modified from the CN-A-K plot of Nesbitt and Young^30^. The addition of M (Mg) was to separate Al-rich kaolinite/gibbsite – proxies for acid weathering – from Al and Mg-rich chlorite, which is a common diagenetic-metamorphic mineral. The paleosol database was scrubbed of samples representing cover rocks and soils developed on chemical sediments. Ca and Mg were corrected for carbonate and apatite content, and K for K-metasomatism. For the detailed methodology, see supplementary note 3. The mineral composition (Fig. S1) of mudstones in drill core LST-12 (Francevillian Group, Gabon) and OPH (Zaonega Formation, Russia) was determined by X-ray diffractometry using a Bruker D8 Advance diffractometer with Cu *K*α radiation and a LynxEye positive sensitive detector. The measured patterns were interpreted and modeled using the Rietveld algorithm-based program Topaz. The relative error of quantification is better than 10% for major phases (>5 wt%) and better than 20% for minor phases (<5 wt%). For the geological setting, location and description of the successions see ref. ^60^ for core LST-12, and refs. ^61–63^ for the OPH core.

Phosphate equilibrium adsorption experiments onto three clay minerals (kaolinite, illite, and montmorillonite) were performed at the following phosphate concentrations: 1, 5, 10, 15, 20, 25, and 30 μM. A 5-mM Na_2_HPO_4_ stock solution was prepared by dissolving Na_2_HPO_4_ salt (ACS certified, Fisher Scientific) into MQ water. Solutions of different phosphate concentration were prepared by diluting the stock solution with a 0.01-M NaCl or 0.56-M NaCl solution to a total volume of 50 ml. Then, 5 ml of solution was pipetted to analyze the initial phosphate concentration. After that, 45 mg of clay was added into solution to make a 1-g/L solid suspension. After agitation, the solution pH of each experiment was adjusted to 4, 6, and 8 separately using 0.1 M HCl and 0.1 M NaOH. The solution pH was consistently maintained during the adsorption period. Once equilibrium was reached, 5 ml of clay suspension was pipetted and filtered through a 0.2-µm filter. The filtrate and the initial 5 ml solution were acidified for ICP-MS analysis. All experiments were performed in duplicate.

Phosphate dynamic adsorption experiments were performed in a 500-ml beaker. For simulation of freshwater conditions, 400 ml of 0.01 M NaCl solution was added to a glass beaker. Then, 0.4 mL of 5 mM Na_2_HPO_4_ stock solution was added to make a 5-μM phosphate solution. A magnetic stir bar was added to keep the solution mixing during the whole experimental period. Once well mixed, a 5-ml aliquot was taken for initial phosphate concentration analysis. After, the solution pH was adjusted to 4 with small aliquots of 0.1 M HCl and 0.1 M NaOH, mimicking freshwater conditions, and 0.395 g of kaolinite was added into the beaker to make a 1 g/L clay suspension. The adsorption of phosphate onto clays under the freshwater condition lasted 24 h and during this process, the pH was maintained at 4 using 0.1 M HCl and 0.1 M NaOH solution. After 24 h, a 5-ml aliquot was syringed out and filtered through a 0.2 µm filter for ICP-MS analysis. Then, the solution pH was adjusted to 8 by a 0.1-M NaOH solution and 12.87 g of NaCl was added into the beaker at the same time to adjust the ionic strength to 0.56 M, representing marine water conditions. Once the pH stabilized, a 5-ml aliquot was collected for phosphate concentration analysis at *t* = 0. The whole marine condition adsorption process took 2 days and the time intervals of sample collection were *t* = 0, *t* = 1 h, *t* = 2 h, *t* = 4 h, *t* = 8 h, *t* = 12 h, *t* = 1 d, and *t* = 2 d. During the experiment, the reaction beaker was covered by parafilm to prevent possible solution evaporation. Same procedure was applied to Al-hydroxide and Fe-oxide. The phosphate concentration in these samples was analyzed by ICP-MS. Detailed experimental information could also be found in Hai et al.^64^.

The cyanobacterium *Synechococcus* sp. PCC7002 was used as an analog strain in the growth experiment to analyze its response to the addition of P-bearing kaolinite clay. This axenic strain was incubated in a modified A + growth media with a limited phosphate concentration of 5 µM. All cultures were grown on a shaker in a designated growth chamber that was set at 30 °C with constant halogen lighting. A sterile 1-L Erlenmeyer flask with 550 mL of modified A+ growth media was initially inoculated from an axenic PCC 7002 culture growing on an agar plate. The growth of the microbial culture was monitored by optical density at 750 nm (OD 750 nm) on a daily basis after inoculation. The growth curve started at day 0 when the OD 750 nm absorbance was above 0.15. A triplicate of 1 mL cultures was extracted sterilely from the flask each day for Chlorophyll-a concentration analysis as an indicator of cell growth from day 0. When it was certain that the culture reached the stationary phase, at which time the Chlorophyll-a concentration stopped increasing at ~0.680 µg/mL, the culture was further divided into three 150 mL sub-cultures in 500 mL Erlenmeyer flasks. Immediately after sub-culturing, 150 mg and 300 mg of P-bearing kaolinite were added into two of the three sub-cultures to make a 1 g/L and 2 g/L kaolinite suspension, respectively. The last sub-culture was the control where no clay was added. All three cultures were continuously examined for cell growth by Chlorophyll-a concentration until day 14, when there was a clear cell density difference among three sub-cultures (Fig. 4). Both optical density at 750 nm and chlorophyll-a concentration was measured on a Beckman Coulter DU®520 UV/VIS spectrophotometer. The chlorophyll-a concentration was lastly converted to cell density using a correlation that was established from a standard growth curve, which was systematically quantified by both chlorophyll-a concentration and cell density that was measured on an Attune NXT acoustic focusing flow cytometer (Fig. 4).

## Supplementary information

Supplementary Information

Description of Additional Supplementary Files

Supplementary Data 1

Supplementary Data 2

Supplementary Data 3

## Data Availability

The authors declare that the main data supporting the findings of this study are available in the following Data Repository link: 10.17632/dmhfrympsr.1.

## Code availability

Code available at 10.5281/zenodo.4641971.
