# Supplementary material for: The kaolinite shuttle links the Great Oxidation and Lomagundi events
Source: Nat Commun. 2021 May 19;12:2944. doi: 10.1038/s41467-021-23304-8 (PMC8134571; doi:10.1038/s41467-021-23304-8)
Supplement: Supplementary file 1 — Supplementary Information [file 41467_2021_23304_MOESM1_ESM.pdf]

Supplementary Information

The kaolinite shuttle links the Great Oxidation and Lomagundi events

Weiduo Hao*[1], Kaarel Mänd[1,2], Yuhao Li[1], Daniel S. Alessi[1], Peeter Somelar[2], Mathieu Moussavou[3], Alexander E. Romashkin[4], Aivo Lepland[2,5,6], Kalle Kirsimäe[2], Noah J. Planavsky[7], Kurt O. Konhauser[1]

[1]Department of Earth and Atmospheric Sciences, University of Alberta, Edmonton, AB T6G 2E3, Canada

[2]Department of Geology, University of Tartu, 50411 Tartu, Estonia

[3]Department of Geology, University of Masuku, 943 Franceville, Gabon

[4]Institute of Geology, Karelian Science Centre, 185610 Petrozavodsk, Russia

[5]CAGE—Centre for Arctic Gas Hydrate, Environment and Climate, Department of Geosciences, UiT The Arctic University of Norway, 9037 Tromsø, Norway

[6]Geological Survey of Norway (NGU), 7491 Trondheim, Norway

[7]The Department of Geology & Geophysics, Yale University, New Haven, CT 06511, USA

Supplementary Note 1 – P distribution in solution and suspended sediments in modern rivers

Compared to N and C that have a virtually unlimited atmospheric source, the supply of P to the oceans derives almost entirely from continental weathering. It reaches seawater via riverine transport in various forms, i.e., dissolved inorganic P, dissolved organic P, or particulate P associated with river suspended sediments (SS). A large portion of P in the riverine reservoir occurs in different phases of SS, including P absorbed onto Fe-mineral particles, carbonates, in clay mineral interlayers, and detrital P minerals. The concentration of dissolved free P in rivers is generally low; however, the fractions of P that exist as exchangeable or adsorbed phases on river SS can easily be released into the water column once aqueous environmental conditions change. In this section, we review P phases in modern river SS (especially bioavailable P) to cast light on ancient riverine shuttling of P into the oceans.

Sequential extraction has been widely applied to determine the speciation of elements in sediments. We selected the literature that applied sequential extraction to river SS to determine the portion of bioavailable P therein. Due to the inconsistency of sequential extraction techniques found in literature, there are several different definitions of bio-available P. For example, Dorich et al. (1984) determined bioavailable P by culturing *S. capricornutum*[1]; Logan et al. (1979) referred to NaOH-extracted P as bioavailable P[2]; and a six-step extraction procedure was utilized by He et al. (2009)[3], who used the sum of exchangeable P, organic P and Fe-bound P to represent bioavailable P. In our compilation of literature data, unless well-defined within a particular study, we used the difference between total P and detrital P to represent bioavailable P.

The inconsistencies in P concentration units are another obstacle in summarizing and comparing literature data. Most studies have applied μM of P per gram of SS (μM/g) or μg of P per gram of SS (μg/g) to represent the fractions of various P phases in river SS. In order to compare

dissolved reactive P among different studies and assess the composition of the riverine P pool, we converted all units into μg of P per liter of water (μg/L) by multiplying the SS P concentration by the riverine SS concentration (g/L). For those published studies that did not provide information on SS concentration, we applied μg of P per gram of SS and % of total particulate P to represent the composition of bioavailable P.

Due to different degrees of anthropogenic impact, the total P concentration as shown in Table S1 varies from 56 μg/L (subglacial rivers)[4] to 28,627 μg/L (Yellow River)[5]. Here, the lower limit is in subglacial rivers representing the lowest anthropogenic impact, while the Yellow River, having the highest total P concentration, is in an area of high population density. The amount of bioavailable P in SS comprises a large portion of total P in SS and the concentration is significantly higher than dissolved P in rivers, indicating that dissolved P only contributes a small percentage of the total P pool in river systems. Indeed, most P exists in bioavailable forms associated with suspended sediments. It should be noted that the subglacial rivers in Svalbard show the lowest total P, bioavailable P and dissolved P[4], which possibly arises due to minimal weathering in the polar environment, as well as P in SS being mainly in the form of detrital P minerals which is difficult for organisms to extract.

Table S1: Compilation of P concentrations in river suspended sediment (SS) and the dissolved phase.

	Bioavailable P in SS	Total P in SS	Dissolved P	% of dissolved P	Reference
Amazon River (Brazil)	133.2μg/L	216.9μg/L	9μg/L	3.98%	Chase and Sayles (1980)[6]
Amazon River (Brazil)	119.6μg/L	141.2μg/L	12μg/L	7.83%	Berner and Rao (1994)[7]
Yamuna River (India)	48.5-81.1μg/L	56.4-103.7μg/L	No data	No data	Chakrapani and Subramanian (1995)[8]
Great lakes tributaries (US)	784.2μg/L	856.2μg/L	No data	No data	Depinto et al. (1981)[9]
Black Creek Watershed (US)	900.5μg/g	2060μg/g	No data	No data	Dorich et al. (1984)[1]
Changjiang River (China)	1096.2μg/L	3669.2μg/L	65.7μg/L	1.76%	He et al. (2009)[3]
Subglacial rivers (Svalbard)	0.5μg/L	56μg/L	below detection limit	<0.01%	Hodson et al. (2004)[4]
Yellow River (China)	846.3-4654.7μg/L	5204.9-28627.0μg/L	4.9-22.7μg/L	0.079%-0.094%	He et al. (2010)[5]
Streams draining into Lake Erie (US)	234.1μg/L	757.3μg/L	135.8μg/L	15.20%	Logan et al. (1979)[2]
Yarra River (Australia)	77-81% of total particulate P	No data	No data	No data	Sinclair et al. (1989)[10]
Yellow river (China)	27.42% of total P	No data	0.11% of total P	0.11%	Yao et al. (2016)[11]
Jiulong River (China)	100-113.2μg/L	114.3-141.8μg/L	54.8-47.2μg/L	27.11%-22.52%	Lin et al. (2013)[12]
Two Lake Erie tributaries (US)	23-27% of total P	No data	No data	No data	Stone and English (1993)[13]
Kleine Aa catchment (Switzerland)	43-66% of total particulate P	No data	No data	No data	Pacini and Gachter (1999)[14]
Upper Brisbane River (Australia)	517μg/g	909μg/g	No data	No data	Kerr et al. (2011)[15]
Patuxent River (US)	93.7-94% of total particulate P	No data	No data	No data	Jordan et al. (2008)[16]
Black Creek Watershed (US)	94-111.7μg/L	470.7-477.7μg/L	46.7-54.5μg/L	9.03%-10.24%	Dorich et al. (1980)[17]

Supplementary Note 2 – The Francevillian Group and the Zaonega Formation

Some of the earliest phosphorites in the world formed during, and directly following, the LE[18]. Here we present two examples of P-rich sections of the time, the Francevillian Group in Gabon and the Zaonega Formation in the Onega Basin of NW-Russia. In addition to hosting abundant apatite $[(Ca,Mg,Sr,Na)_{10}(PO_4,SO_4,CO_3)_6F_{2-3}]$, mudstone intervals in these sections preserve kaolinite, suggesting that it was a common clay phase in P-rich sediments of the LE period (Fig. S1, S2, S3).

The Francevillian Group (FG) is a Paleoproterozoic succession of organic-rich mudstones intercalated by dolostones, sandstones, and cherts[19]. The age of FG deposition is constrained by U-Pb zircon dates of 2083 ± 6 Ma in welded ignimbrite tuffs in the upper part of the succession[20] and a tentative, disconcordant 2191±13 Ma age of zircons from granitoids of the N'goutou Complex intruding the lower part of the succession[21]. This age interval is consistent with Sm-Nd dates of the shale clay fraction between ~2200 and 2050 Ma[22]. The upward change from isotopically heavy $\delta^{13}C$ values (as high as +10) to normal ($\delta^{13}C$ values near zero) found in the FG succession has been interpreted to record the termination of LE[23-25]. The carbon, molybdenum, and sulfur isotope composition, as well as the iron speciation and trace metal content of the upper FG, has been inferred to reflect a transition from oxygen-rich to oxygen-poor conditions in the ocean-atmospheric system[23,25,26]. Mixed-layer smectitic and kaolinite clay minerals in the succession have been found to be exquisitely preserved, having undergone little diagenesis and no metamorphic alteration[27,28]. Low levels of post-sedimentary alteration are further indicated by the preservation of putative macrofossils of the Francevillian Biota[29].

The drill core LST-12 from the Lastoursville sub-basin[30] comprises, in the lower part, thinly-interbedded relatively shallow-water shales and dolomarls belonging to the Franceville FB-

C Formation that contains up to ~3 wt.% of kaolinite. It then transitions to pink dolostones with abundant pyrobitumen veining that are rich in apatite (up to ~25 wt.%; Fig. S1, S2, S3). The upper part of LST12 records basinal deepening below the effective storm wave base, leading to the deposition of organic-rich shales of the Franceville FD Formation that again host kaolinite (up to ~3 wt.%).

The Zaonega Formation (ZF) is a ~1500-m-thick mixed sedimentary-volcanic succession that overlies the ^{13}C-enriched evaporites and carbonates of the Tulomozero Formation, which is one of the type sections of the LE[31]. The ZF itself consists of graded greywackes and organic-rich mudstones interbedded with carbonates and mafic volcanic rocks. The end of the LE positive $\delta^{13}C$ excursion, constrained at ~2060 Ma in Fennoscandi[32], is recorded in the lower part of the ZF. A Re-Os age of ~2050 Ma was reported by Hannah et al. (2008) from upper ZF mudstones[33]. On the other hand, a tuff layer in the lower/middle ZF yielded a 1982±4.5 Ma zircon Pb-Pb age[34] and several cross-cutting sills and overlying lava flows have been dated between ~1919–1988 Ma[34-38], leaving the age of the ZF controversial. Carbon, sulphur, molybdenum, and uranium isotope ratios, as well as rare earth element composition in the ZF have alternatively been interpreted as reflecting the collapse of an oxygenated ocean-atmospheric system following the end of the LE[31,39-41], or the continuation of oxygenated conditions[42-46].

The Onega Parametric Borehole (OPH), drilled in the southern part of the Onega Basin (62.1559 N, 34.4073 E, Ref. 47), intersects a basin-wide layer of apatite (up to ~6 wt.% in the OPH drill-core) hosted in highly organic-rich mudstones (up to 60 wt.%) and carbonates (Fig. S1; Ref. 48). This layer occurs at the transition from a deeper-water anoxic mudstone facies to a possibly shallower and sub-oxic carbonate-rich depositional setting[44]. The ZF has undergone greenschist-facies metamorphism that commonly results in the conversion of kaolinite to illite or

sericite. XRD modelling results suggest that the OPH section shown here retains up to ~2 wt.% of kaolinite in mudstones overlying the P-rich zone. However, the identification of kaolinite in the Zaonega Formation remains tentative, given the low concentrations of kaolinite, and the thermal history of the rocks.

Both of these sedimentary basins have recently been interpreted as hydrographically restricted, complicating efforts to use their geochemistry for the purpose of global biogeochemical inferences[30,43,44,49-51,56]. However, kaolinite generation occurs mostly in terrestrial weathering environments and is carried to marine sediments through rivers. Hence, the potentially restricted nature of these basins is not likely to affect kaolinite accumulation mechanisms. Furthermore, if these restricted basins were less affected by upwelling of P-rich deep waters, kaolinite P shuttling is likely to have an even more outsized effect on P accumulation.

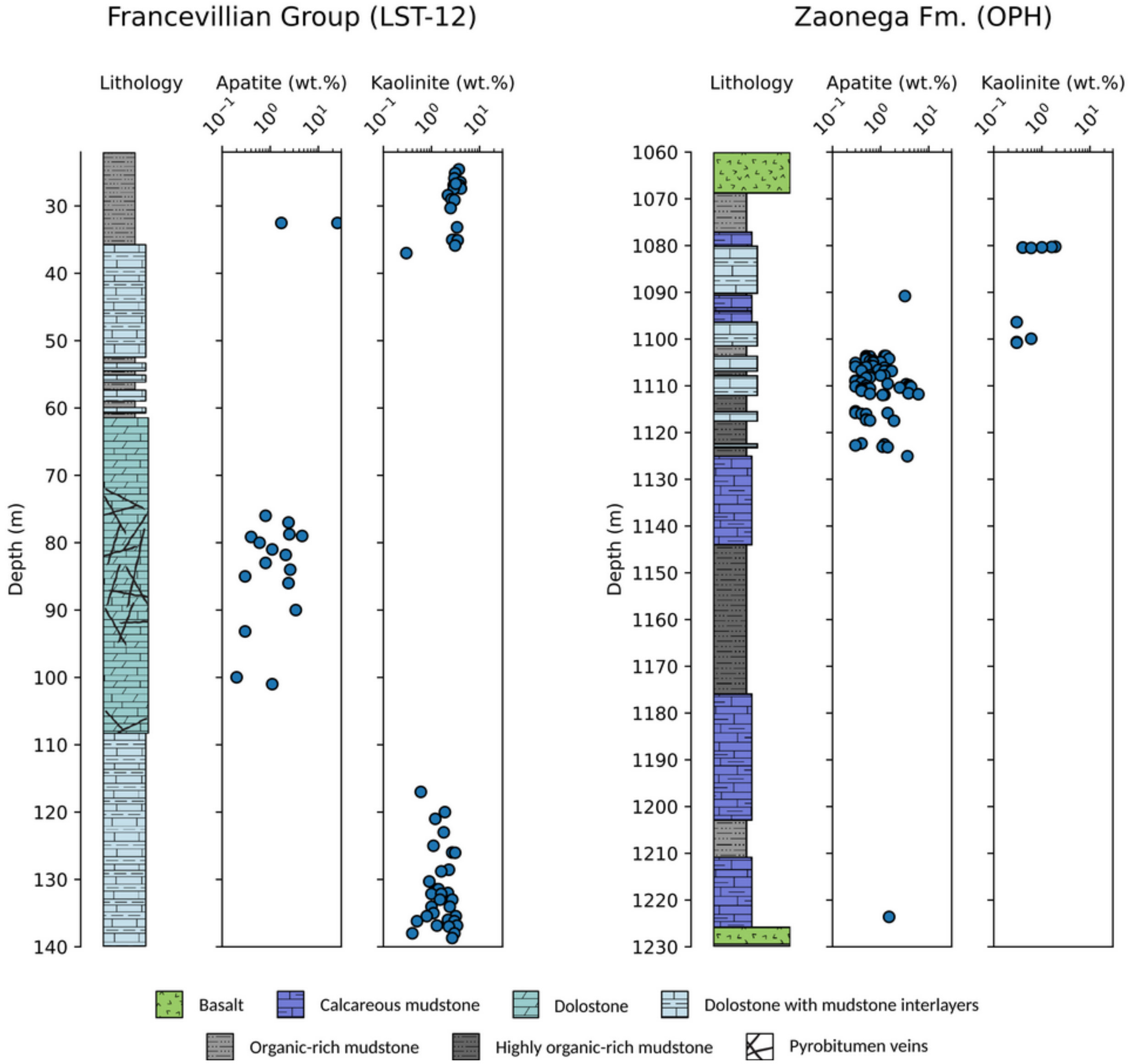

Fig. S1: X-ray diffraction (XRD) quantification evidence for kaolinite and apatite in post-GOE shales. Lithological columns with associated legend show the simplified lithological context for XRD samples in the ~2.2–2.05 Ga upper Francevillian Group in Gabon (drill core LST-12) and the ~2.0 Ga Zaonega Formation in Russia (drill core OPH).

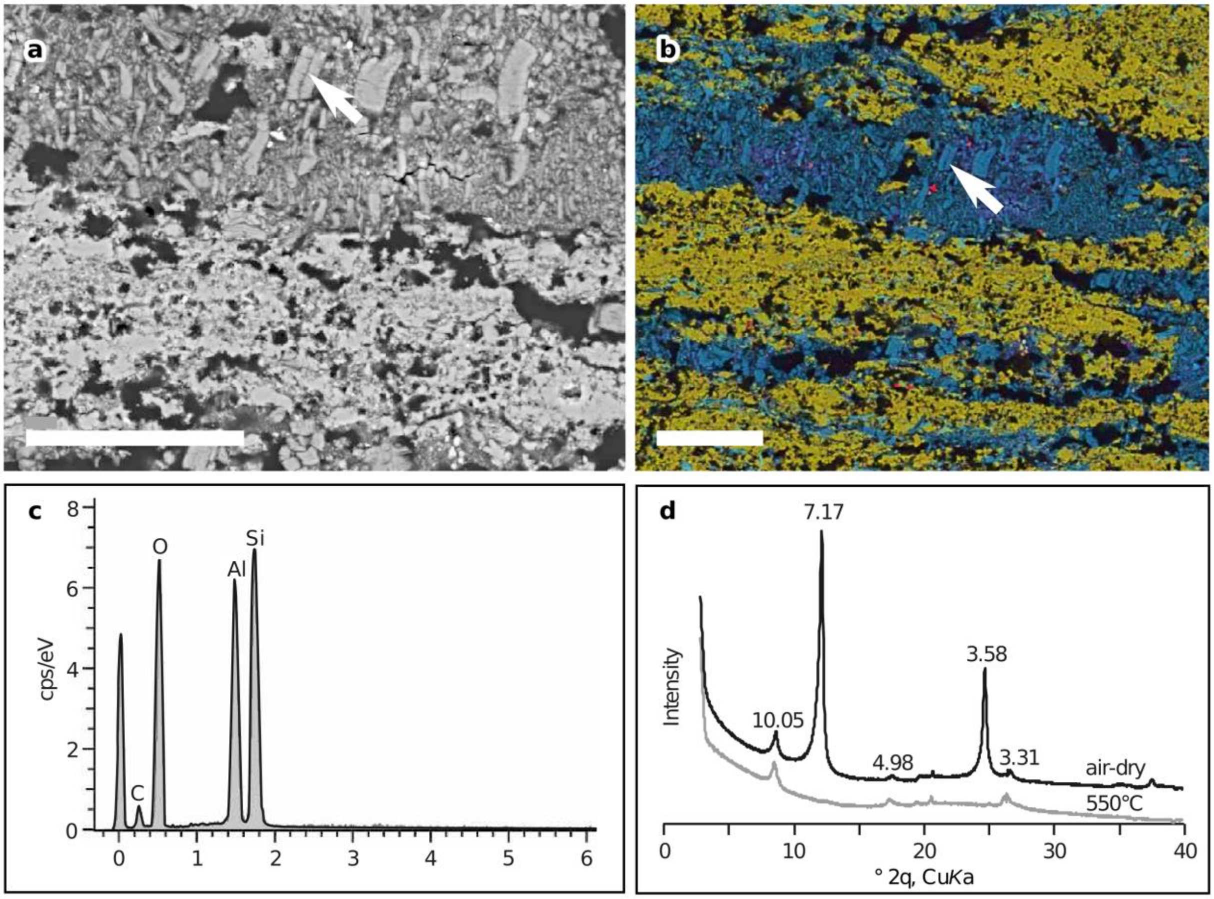

Fig. S2: Kaolinite XRD spectra in Francevillian shales. (a-b) Scanning electron micrographs of polished slabs from a Francevillian black shale outcrop in the Lastrousville train station, Francevillian FD Formation (sample FR17-FD2b). Scale bars are 100 μm across. (a) Back-scattered electron image; arrow shows an example of a kaolinite crystal. Scanning electron microscopy was done on a Zeiss EVO MA15 SEM at the University of Tartu. (b) Larger view of the same area overlain with relative abundances of Al (blue), Ti (red), S (purple), and Si (yellow), mapped using an Oxford AZTEC-MAX energy-dispersive spectrometer attached to the SEM. (c) Energy-dispersive X-ray spectrum of the above sample, collected near the arrow on panels a and b. (d) Characteristic XRD spectra of the oriented clay fraction (<2 μm) of the above sample. The clay fraction preparations were measured at an air-dry state and after heating at 550˚C for 1 hour for phase identification. Heating of the kaolinite above 500˚C causes its decomposition and kaolinite peaks disappear while overlapping chlorite basal reflections remain in the XRD pattern.

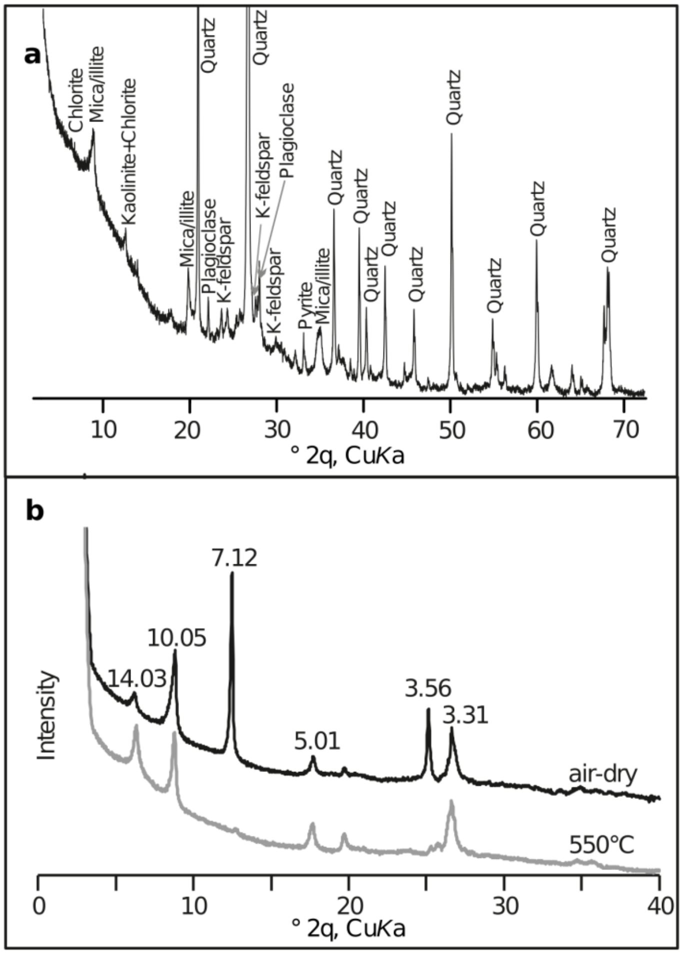

Fig. S3: Whole rock (a) and oriented clay fraction (b) XRD spectra of a mudstone in the LST-12 drill core (sample #5, depth, 35.1 m).

Supplementary Note 3 - The compilation of paleosol data

The acidity of weathering is expressed in paleosol mineralogy through the abundance of Al-rich components, such as kaolinite or gibbsite. However, the majority of published paleosol mineralogy data do not include quantification of phyllosilicate minerals. Furthermore, kaolinite is not often well-preserved over geologic time – the prevalence of K-metasomatism in old terranes[52] means that kaolinite is often converted to illite[53], sericite[54,55], or illite-sericite[56], depending on the composition of the altering fluid. Alternatively, under high burial temperatures and pressures, SiO_2-rich fluids can transform kaolinite into pyrophyllite[57] or kyanite[58].

In this situation, an indication of the original importance of acid weathering products in paleosols must be gleaned from their major element chemistry. The evolution of rock chemical composition during weathering and the relative weathering intensity are commonly expressed using a CN-A-K (CaO+Na_2O, Al_2O_3, K_2O) ternary plot and the related Chemical Index of Alteration (CIA)[59]. In this case, CaO is corrected for potential contributions from apatite and calcite (see Methods below). While this type of plot neatly illustrates the progression of weathered rocks towards more Al-rich compositions, it lacks resolution regarding the acidity of weathering because acid weathering products like kaolinite/gibbsite plot together with chlorite, as both contain Al but are devoid of Ca, Na and K. Since most Proterozoic-Archean paleosols have been through at least lower greenschist-facies metamorphism, they are typically rich in chlorite (often forming at the expense of Fe-Mg smectite clay minerals developed in weathering of mafic parent rocks). Also, the classical CN-A-K plot and CIA values are biased towards lower apparent weathering intensity by common K-metasomatism[52,60].

To estimate the relative pre-diagenesis/metamorphic kaolinite content in weathered materials, we introduce the CaO^*+Na_2O+MgO^*, Al_2O_3, K_2O^* (CNM-A-K) plot, which is capable

of distinguishing kaolinite and chlorite (Fig. S4). The location of common aluminosilicate phases on the CNM-A-K plot shows that data near the top corner of the ternary plot suggest an important contribution from kaolinite and/or gibbsite, in relation to other aluminosilicate minerals. In a compilation of 10 relatively well-preserved and well-characterized paleosols from the late Archean through the Paleoproterozoic (Table S2), those in the GOE and LE period (Hokkalampi, Hekpoort, and Drakenstein paleosols; Refs. 61,62) plot generally higher on the CNM-A-K plot than those older than 2.4 Ga (Mt. Roe, Kuksha, and Cooper Lake; Refs. 63-66) or those younger than 2.0 Ga (Beaverlodge Lake, Flin Flon, Baraboo; Refs. 67-70), indicating an original composition possibly more rich in acid weathering products. This trend is not likely an artifact of the differing latitude at which these paleosols formed as calculated paleolatitudes do not correspond to the above trend (Supplementary Data 2). The exceptions to the overall trend are the Archean Mt. Roe and Paleoproterozoic Ville Marie paleosols. Al-rich compositions in Mt. Roe[56] may attest to occasionally intense weathering regimes under a high-CO_2 Archean atmosphere (alongside some samples from the Kuksha paleosol[63]). Alternatively, Teitler et al. (2015) interpreted the Mt. Roe paleosol to have formed in the presence of significant amounts of oxygen (i.e., under similar intensive weathering conditions as GOE paleosols)[56]. The Ville Marie paleosol displays a weathering regime more akin to pre-2.4 and post-2.0 Ga paleosols, but its age is poorly constrained to between 2.44 to 2.21 Ga[71]; as such, it does not necessarily argue against high weathering intensity post-2.4 Ga.

These data support the hypothesis of Konhauser et al.[10] that the early Proterozoic terrestrial environment was subjected to intense acid weathering due to the oxidation of exposed pyrite. Similarly, siderite weathering may have increased atmospheric CO_2 levels, boosting overall

weathering rates[72]. This pulse of intense, acidic weathering resulted in the enhanced generation of kaolinite.

We further investigated P weathering fluxes across our paleosol database, by using τ values (τP_2O_5), which track the loss or gain of P compared to the protolith by normalization to an immobile element, e.g., Ti:

$$\tau P_2O_5 = \{[P_2O_5/TiO_2 \text{ (sample; wt.\%/wt.\%)}] / [P_2O_5/TiO_2 \text{ (protolith; wt.\%/wt.\%)}]\} - 1.$$

We find that paleosols deposited between 2.4–2.0 Ga display on average more extreme P loss (lower τP_2O_5 values; Fig. S5), possibly reflecting increased apatite dissolution under acidic weathering regimes of the Paleoproterozoic[73].

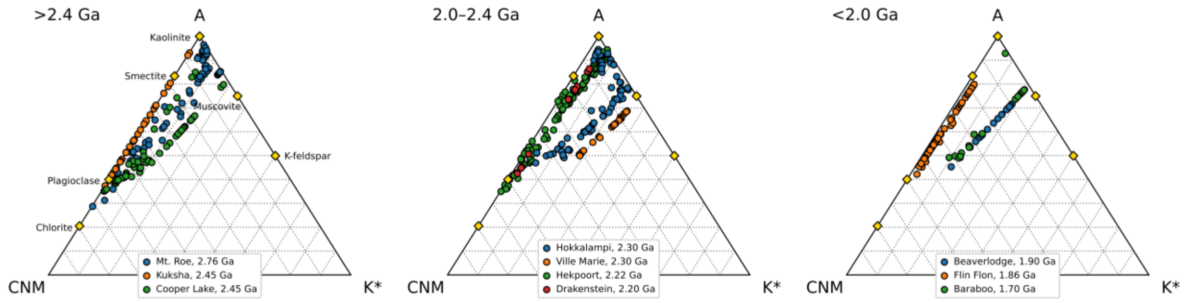

Fig. S4: Major element composition of Late Archean to Paleoproterozoic paleosols. (a-c) Ternary CNM-A-K plots illustrate weathering trends of individual paleosols. Element data are presented in molar proportions divided by age brackets. CNM = CaO^* + Na_2O + MgO^*; A = Al_2O_3; K* = K_2O^*. Asterisks denote data corrected for non-silicate contribution and K-metasomatism (see Methods). Yellow diamonds denote stochiometric compositions of common aluminosilicate minerals in igneous/siliciclastic rocks and paleosols.

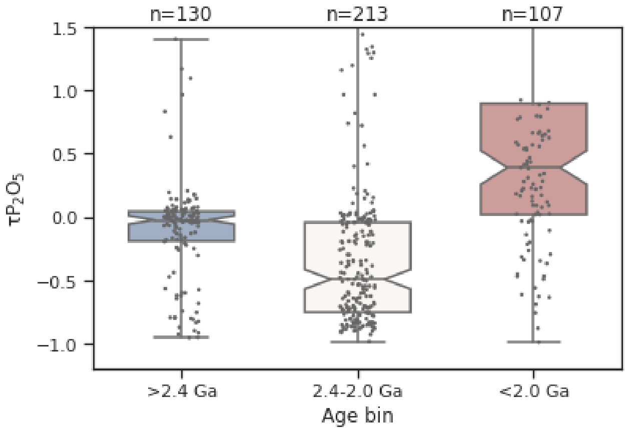

Fig. S5: Phosphorous leaching in Late Archean to Paleoproterozoic paleosols. Negative τP_2O_5 values denote P loss compared to protolith, positive values denote preferential P retention. See Fig. 1 for explanation of figure elements.

Methods

The paleosol chemical database was compiled using 10 published paleosol studies ranging in age from 2.76 Ga to 1.70 Ga (Table S2). The paleosol selection was based on the relative completeness of the profiles, e.g., whether samples of both the protolith and the topmost weathering horizons were available. Samples representing sediments or volcanic rocks covering the paleosol were removed.

The whole rock CaO data were corrected for the contribution of CaO from calcite, dolomite, and apatite, arriving at an estimate of silicate CaO (CaO*). In most cases, the mineralogical composition and/or CO_2 data for paleosols are not available for correction. Therefore, the CaO* content was derived following the method of McLennan et al.[74], assuming reasonable values of Ca/Na ratios in the silicate material. In the first step, the Ca bound in apatite was subtracted using the molar proportion of P_2O_5 from the molar proportion of total CaO. In the next step, CaO* was assumed to reside in plagioclase, hence if the remaining CaO was less than the molar proportion of Na_2O, then that CaO was considered to represent CaO*. However, if the

amount of remaining CaO post-apatite correction was greater than the Na_2O, then CaO* was considered to equal the amount of Na_2O.

MgO in the silicate fraction (MgO*) was calculated based on similar assumptions – that the whole rock MgO data can include MgO in carbonate minerals (mainly dolomite). CaO in the carbonate fraction (whole rock CaO minus CaO* and $CaO_{apatite}$) was conservatively assumed to reside entirely in dolomite. MgO* was derived by subtracting the respective molar proportion of MgO in dolomite, assuming a stoichiometric composition, from whole rock MgO data. The MgO* calculated by this approach represents the minimum value of Mg in the silicate fraction and true MgO* can be higher if calcite occurs instead of dolomite. However, dolomite is commonly present as a secondary phase in paleosols, typically found in the middle to lower parts of the weathering crusts at or below the paleogroundwater table (e.g., Ref. 75). Thus, the possible underestimation of MgO* does not affect the kaolinite estimate in the uppermost highly weathered samples.

During diagenetic and metamorphic processes, the soil clay minerals formed during weathering accumulate K because weathering-derived smectitic and mixed-layer minerals as well as kaolinite readily take up K from diagenetic solutions, even in cases where the protolith is depleted in K[60]. It, therefore, becomes important to assess the K content prior to metasomatism (K*) for individual samples by assuming a predicted weathering trend following the procedures described in Fedo et al.[52] and Medaris et al.[68,76]: since K-feldspar weathering lags behind that of plagioclase, the weathering process will conserve the protolith K/Al ratio until the end stages of weathering. Any K values trending higher than expected from this ratio are conservatively assumed to be affected by K-metasomatism. The K* derived in this way represents the minima for those samples that plot on A-K axis of the CN(M)-A-K plot (i.e., have lost all Ca, Na, and Mg during the weathering).

The paleolatitude of the paleosol sites (Supplementary Data 2) was calculated form paleomagnetic pole coordinates at the time of weathering using the approach described in Haile[77]. Northern latitudes of the paleosols current geographic location are given as positive and southern latitudes as negative values. Longitudes are given as positive values measured east from the zero meridian to 360° (Ref 77). The sign of the paleolatitude is positive if the site's position is <90° from the paleopole and negative when distance is >90°. Paleopole locations for stable cratons where the paleosol is located were selected from available data by closest matching age to the estimated time of weathering[78-85].

Data analysis and visualization utilized the *pandas*[86], *python-ternary*[87], *matplotlib*[88], and *seaborn*[89] modules for the Python programming language.

Table S2: Archean–Proterozoic paleosols used to estimate weathering intensity and kaolinite content. For paleolatitude data, see Supplementary Data 2; for geochemical data, see Supplementary Data 1.

Paleosol	Age (Ga)	Location	Paleolatitude (°)	Protolith	Data references
Mt Roe	~2.76	Pilbara Craton, W-Australia	45.08	Basalt	Refs. 65,66,85
Kuksha	2.44-2.50	Imandra-Varzuga Greenstone Belt, NW-Russia	-29.24	Basalt	Refs. 63,83
Cooper Lake	~2.45	Huronian Supergroup, ON, Canada	-13.42	Sediment-hosted mafic dike	Refs. 64,70,81
Ville Marie	~2.44-2.2	Huronian Supergroup, QC, Canada	25.25	Granite	Refs. 71,90
Hokkalampi	~2.44-2.2	Karelian Craton, Finland	27.24	Granite	Refs. 62,86
Hekpoort	<2.224	Transvaal Supergroup, South Africa	-13.90	Basaltic andesite	Refs. 61,84
Drakenstein	~2.22	Transvaal and Olifansthoek Supergroups, South Africa	-10.86	Basaltic andesite	Refs. 83,91
Beaverlodge Lake	<1.931	Northwest Territories, Canada	50.94	Feldspar (±quartz) porphyry	Refs. 74,80
Flin Flon	~1.86	Manitoba, Canada	31.48	Pillowed greenstone	Refs. 67,70,79
Baraboo	~1.7	Wisconsin, USA	51.91	Granite	Refs. 69,82

Supplementary Note 4 - Surface complexation modelling (SCM) of phosphate adsorption onto kaolinite surfaces

At low pH, the surface functional groups of kaolinite are highly protonated, lending the clay a net positive charge. At pH<4.7, kaolinite surfaces are dominated by $\equiv XOH_2^+$ (0.023 mmol/g, representing amphoteric groups such as $\equiv Al\text{-}OH_2^+$ or $\equiv Si\text{-}OH_2^+$) and ≡LH (0.068 mmol/g, representing isomorphic substituted adsorption sites) groups, and both continuously deprotonate with increasing pH (Fig. S6), causing kaolinite to have an increasingly negative surface charge. The amphoteric surface functional group, $\equiv XOH_2^+$, undergoes two deprotonations at pH 4.7 ($\equiv XOH_2^+$ to $\equiv XOH^0$) and at pH 6.5 ($\equiv XOH^0$ to $\equiv XO^-$). The monoprotic ≡LH site has a single deprotonation reaction at a pKa of 9.6 (≡LH to $\equiv L^-$).

The aqueous speciation of phosphate includes four primary species[92]. At pH<2, P is highly protonated as phosphoric acid, H_3PO_4. Three subsequent deprotonation reactions, with *pKa* values at 2.15, 7.20, and 12.35, result in the formation of the species $H_2PO_4^-$, HPO_4^{2-}, and PO_4^{3-}, respectively. At pH<4.7, the predominant aqueous phosphate species is negatively charged ($H_2PO_4^-$) which would be electrostatically attracted to positively charged $\equiv XOH_2^+$ functional groups on the kaolinite surface. As pH increases to 8, the kaolinite surface is dominated by deprotonated $\equiv L^-$ and $\equiv XO^-$ groups that are repulsive to negatively charged $H_2PO_4^-$ and HPO_4^{2-} species, reducing P adsorption.

P adsorption onto kaolinite is best modelled by invoking P adsorption to both ≡XOH and ≡LH sites. In the model, one HPO_4^{2-} anion forms a bidentate mononuclear surface complex with two ≡XOH sites, while the ≡LH sites form a monodentate complex with a HPO_4^{2-} anion. The adsorption constants (K_{P1} and K_{P2}) are provided in Table S3.

Methods

Acid-base titration experiments coupled with surface complexation modelling were performed to determine the protonation/deprotonation behaviour of the clays between pH 4 and 10.5. Based on previous models of clay surface reactivity, two surface functional groups were invoked in the protonation modelling; the protonation reactions used are given in Table S3 (detailed information is provided in Refs. 93,94).

Batch pH edge P adsorption experiments were performed at 10 μM of P and 1 g/L kaolinite. To initiate an experiment, 100 ml of 10 μM phosphate solution was prepared in a 150 ml beaker by diluting a 5 mmol Na_2HPO_4 stock solution. This stock solution was divided into 10 mL aliquots, which were subsequently adjusted to cover a pH range between approximately 3 and 9. The details of the experimental procedure were similar to our previous study[95]. The pH edge experiments were performed in duplicate to ensure the accuracy of results. The phosphate concentration in these samples was analysed by ICP-MS/MS (Agilent 8800) in the Environmental Geochemistry Laboratory at the Department of Earth and Atmospheric Sciences, University of Alberta. Adsorption edge data were modelled using a non-electrostatic surface complexation modelling approach in the software package Fiteql 4.0 (ref 96). Table S3 lists the P surface reactions that are used in the models.

The experimental data were fitted using a single site bidentate adsorption model, a single site monodentate model, a double site bidentate model, and a double site monodentate model (fitting results are shown in Fig. S7, while the schematic models of the four models are shown in Fig. S8). Amongst these models, the double site monodentate model had a poor fit at low pH, while both single site bidentate and single site monodentate models underestimated P adsorption at high

pH. The double site bidentate model adequately described P adsorption data across the tested pH range. Adsorption equilibrium constants derived from the modelling are listed in Table S3.

Table S3: Surface complexation modelling results of P adsorption onto kaolinite.

Reaction	Equilibrium constant	logK1	Site density 1 (mol/g)
Surface protonation reactions			
$\equiv LH \leftrightarrow H^{+} + \equiv L^{-}$	$K_a =$	9.625*	6.789E-5*
$\equiv XOH \leftrightarrow H^{+} + \equiv XO^{-}$	$K_{a-=}$	6.539*	2.305E-5*
$\equiv XOH + H^{+} \leftrightarrow \equiv XOH_2^{+}$	K_{a+}	4.707*	
Phosphate adsorption reactions			
$\equiv LH + HPO_4^{2-} \leftrightarrow \equiv LH{-}HPO_4^{2-}$	$K_{P1} =$	16.06**	
$2\equiv XOH_2^{+} + HPO_4^{2-} \leftrightarrow (\equiv XOH_2^{+})_2{-}HPO_4^{2-}$	$K_{P2} =$	37.46**	
Hydrolysis reactions and sodium complexes			
$PO_4^{3-} + H^{+} \leftrightarrow HPO_4^{2-}$	$K_1 = \frac{HPO4^{2-}}{PO4^{3-}\quad H^{+}}$	12.38***	
$PO_4^{3-} + 2H^{+} \leftrightarrow H_2PO_4^{-}$	$K_2 = \frac{H2PO4^{-}}{PO4^{3-}\quad H}$	19.57***	
$PO_4^{3-} + 3H^{+} \leftrightarrow H_3PO_4$	$K_3 = \frac{H3PO4}{PO4^{3-}\quad H}$	21.72***	
$PO_4^{3-} + H^{+} + 2Na^{+} \leftrightarrow Na_2HPO_4$	$K_4 = \frac{Na2HPO4}{PO4^{3-}\quad 2}$	13.32***	
$PO_4^{3-} + 2Na^{+} \leftrightarrow Na_2PO_4^{-}$	$K_5 = \frac{Na2PO4^{-}}{PO4^{3-}\quad Na}$	2.59***	
$PO_4^{3-} + 2H^{+} + Na^{+} \leftrightarrow NaH_2PO_4$	$K_6 = \frac{NaH2PO4}{PO4^{3-}\quad 2}$	19.87***	
$PO4^{3-} + H^{+} + Na^{+} \leftrightarrow NaHPO_4^{-}$	$K_7 = \frac{NaHPO4^{-}}{PO4^{3-}\quad H^{+}\quad Na^{+}}$	13.45***	
$PO4^{3-} + Na^{+} \leftrightarrow NaPO_4^{2-}$	$K_8 = \frac{NaPO4^{2-}}{PO4^{3-}\quad Na^{+}}$	1.430***	

Note: * are values from Ref. 94; ** are values derived from this study; *** are values from Visual MINTEQ version 3.1.

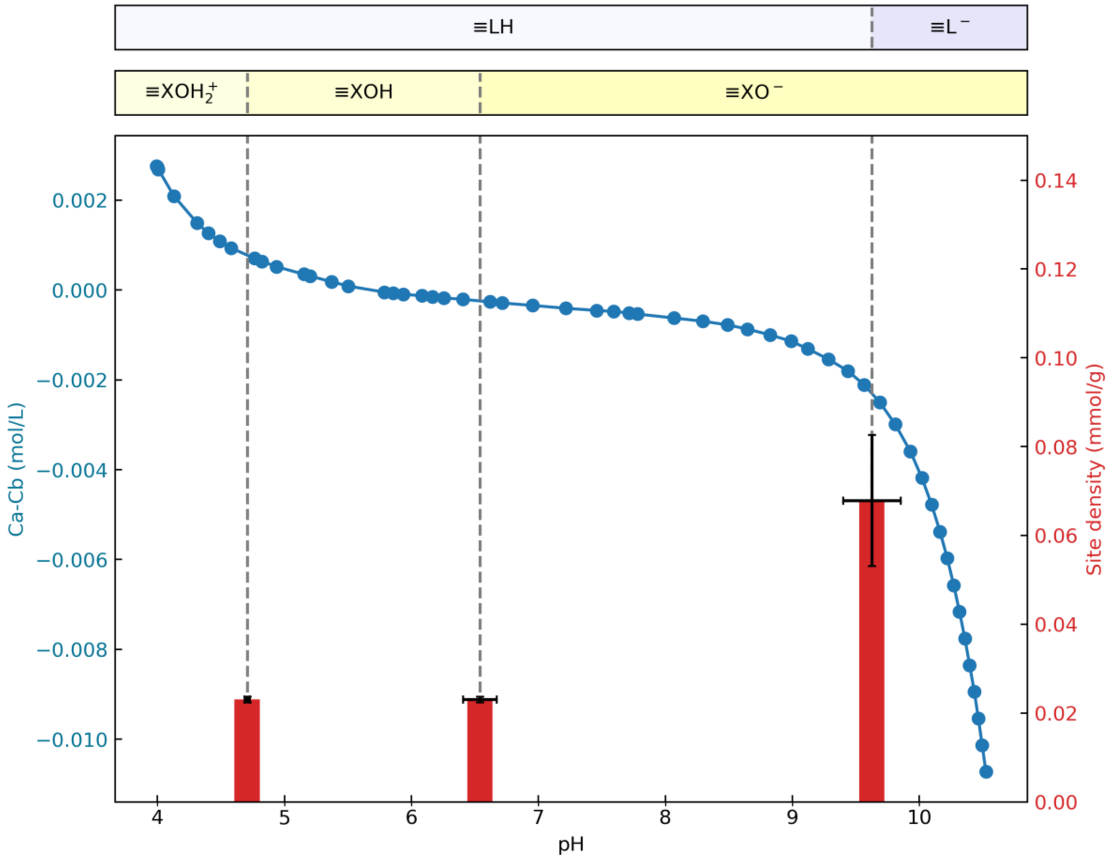

Fig. S6: Titration and surface complexation modelling results of kaolinite. Blue dots and line are raw titration data (C_a-C_b as a function of pH; C_a-C_b is the subtraction of added acid and base concentration, representing the net protons added). Red bars are the pK_a values of the two surface functional groups plotted with the site concentration of two groups: the left and middle red bars represent the ≡XOH group (site concentration is 0.023mmol/g, left y axis) with the protonation to $≡XOH_2^+$ at pH 4.7, and the deprotonation to $≡XO^-$ at pH 6.5; the rightmost red bar is the ≡LH group (site concentration is 0.023mmol/g, left y axis) with the deprotonation pH at 9.6. The blue and yellow bars on top of the figure represent dominant surface site species as a function of pH: $≡XOH_2^+$ dominates at pH<4.7; ≡XOH dominates from pH 4.7 to 6.5, and $≡XO^-$ is the main species at pH>6.5. The ≡LH group dominates at pH<9.6, and $≡L^-$ is the main species at pH>9.6. Error bars represent the ±1 standard deviation of triplicates.

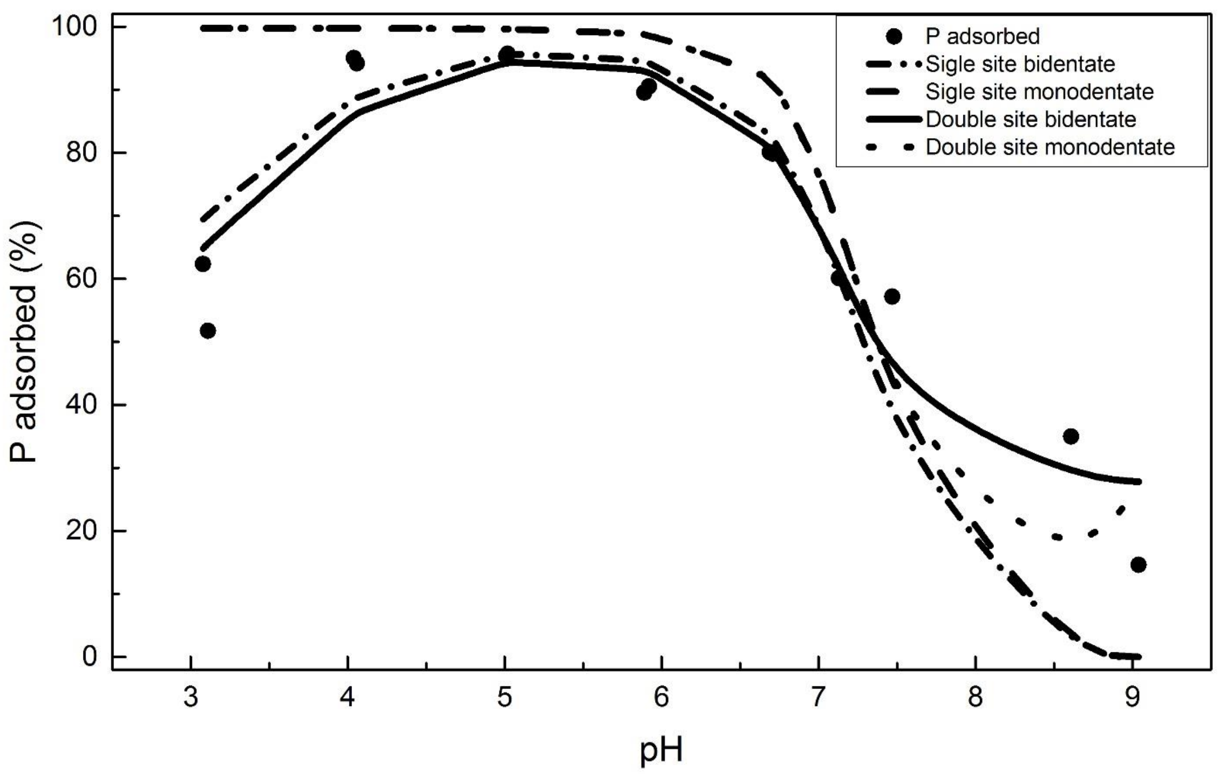

Fig. S7: Fitting of phosphate adsorption pH edge data by different surface complexation models. Closed circles represent experimental data; lines represent the fitting of data by different models.

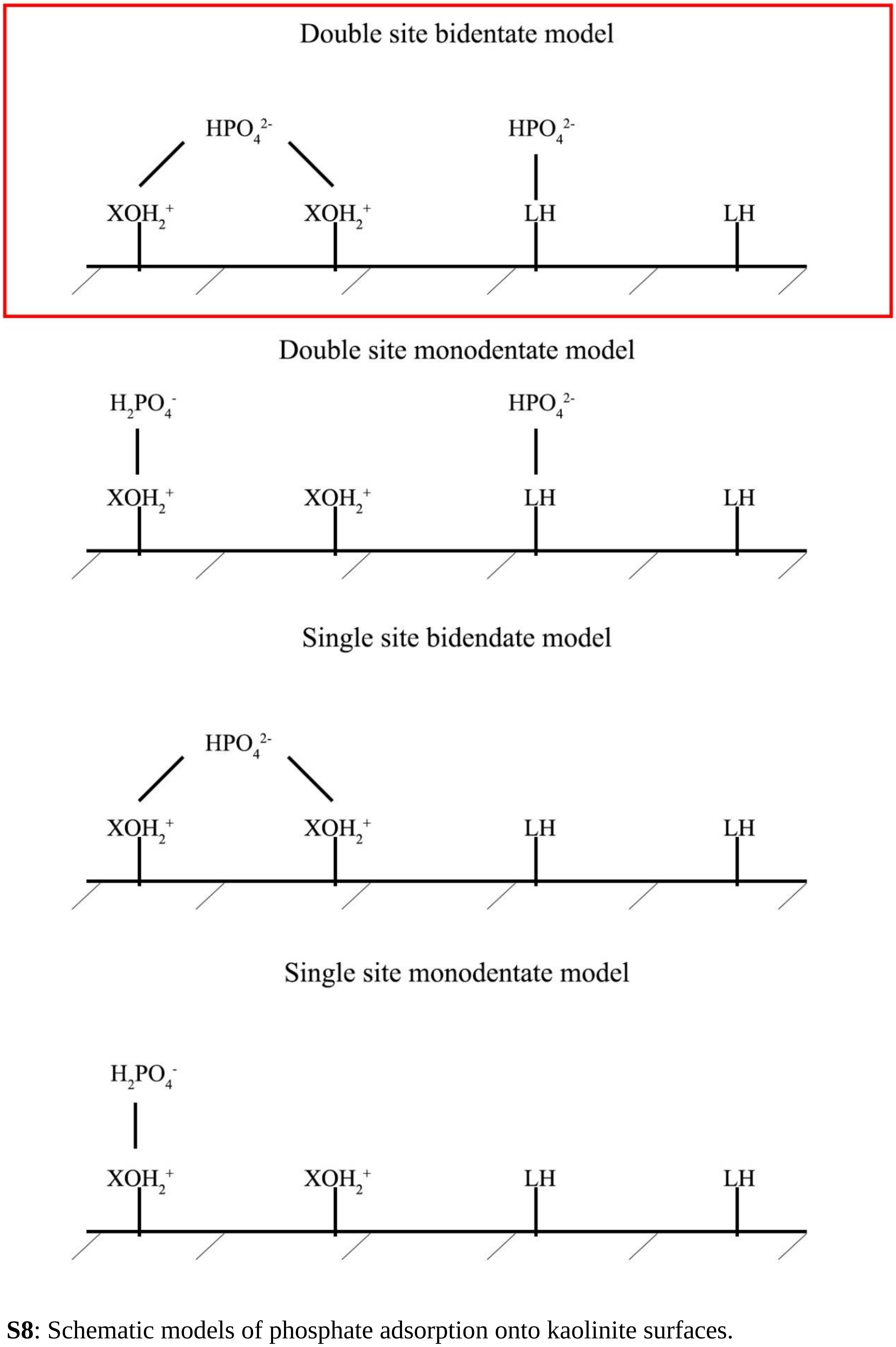

Fig. S8: Schematic models of phosphate adsorption onto kaolinite surfaces.

Supplementary Note 5 - P adsorption capacity of montmorillonite and illite, and the desorption of P from mixtures of weathering products

Phosphate equilibrium adsorption experiments onto montmorillonite and illite were performed as a function of P concentration. The experimental procedure is the same as that used to derive the kaolinite equilibrium adsorption experiments (specific experimental procedure is given in the main text, Methods section). The adsorption isotherms of P onto montmorillonite and illite are shown in Fig. S9. P adsorption onto illite surfaces shows no significant difference at a low initial P concentration for the tested three conditions. However, with an increase in initial P concentration, illite and montmorillonite at the freshwater condition (pH 4, ionic strength (IS)=0.01 M) show higher adsorption capacities than at the other two aqueous conditions. The marine condition has the lowest P adsorption capacity. Montmorillonite displays differences in P adsorption capacity even at low initial P concentrations, with the highest P adsorption at pH 6 and IS=0.01. Throughout the P concentration range, the pH 6 and IS=0.01 condition has the highest P adsorption capacity compared to the other two conditions, while the marine water condition has the lowest P adsorption capacity.

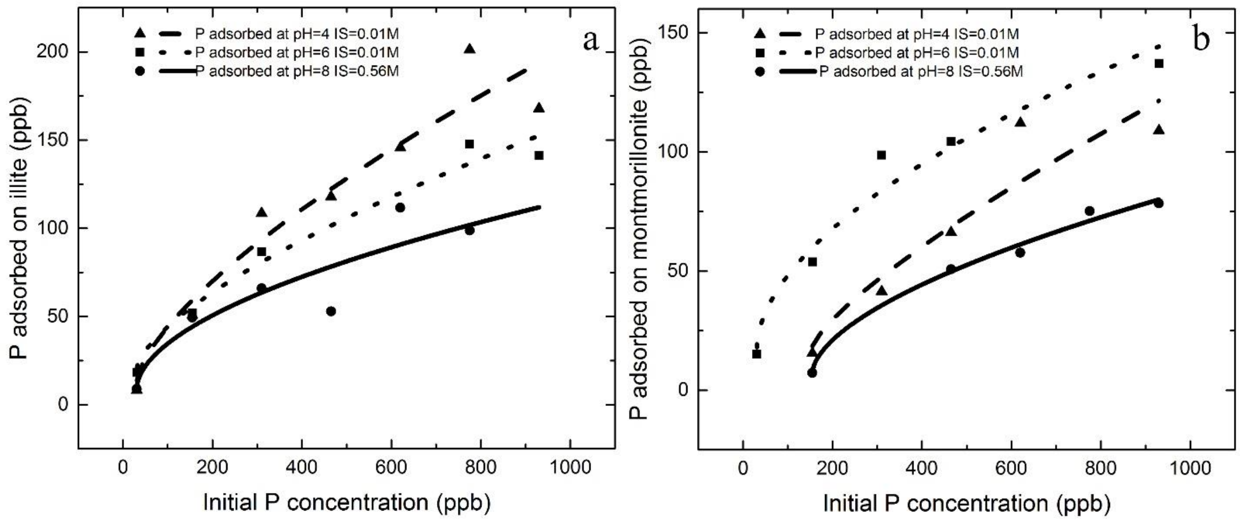

Fig. S9: Phosphate adsorption isotherms for (a) illite and (b) montmorillonite.

Due to the heterogeneity of weathering in natural environments, the minerals comprising the weathered product can range from illite to kaolinite and Fe-oxides. To test the P desorption properties of common mixtures of weathering products when transitioning from freshwater to marine conditions, dynamic adsorption experiments of phosphate on a mixture of kaolinite, illite and Fe-oxide surfaces were performed. The experimental procedure was the same as that used to derive desorption of P from individual surfaces of kaolinite, Al-oxides and Fe-oxides (see Fig. 2 in the main manuscript). The results showed that both a mixture of kaolinite, illite and Fe-oxides, and a mixture of illite and Fe-oxides can release approximately 30% of P when aqueous conditions change from freshwater to marine (Fig. S10). By comparison, the mixture of kaolinite, illite, and Fe-oxides desorb slightly more P than the mixture of illite and Fe-oxides, highlighting the higher desorption of P from kaolinite.

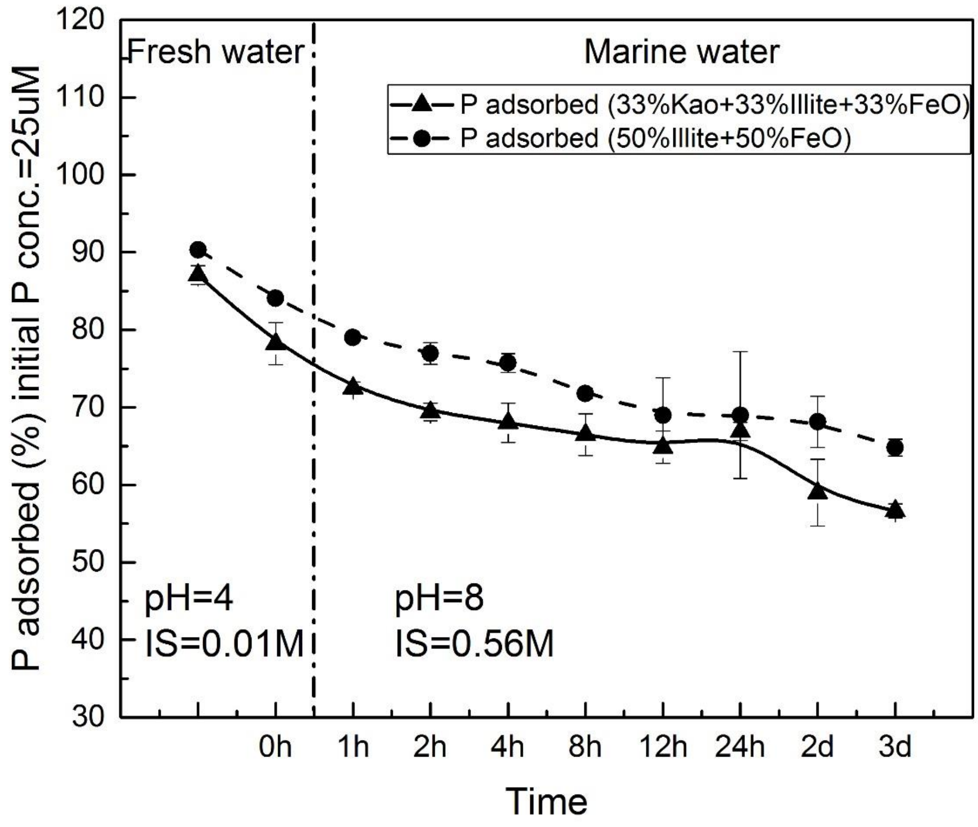

Fig. S10: A comparison between the desorption of P from a mixture of kaolinite, Fe-oxide and illite surfaces, and a mixture of illite and Fe(III) oxide surfaces when transitioning from freshwater to marine conditions. Freshwater conditions were simulated at pH 4 and ionic strength=0.01 M solution, while marine conditions are pH 8 and ionic strength=0.56 M. Error bars represent the ±1 standard deviation of replicates.

Supplementary Note 6 – Increased kaolinite abundance in association with the Palecene-Eocene Thermal Maximum (PETM)

The PETM occurred approximately 56 Mya, when global temperature increased by around 5–8°C[97] as determined by a decrease in O isotope of benthic foraminifera[98]. The PETM was likely due to massive CO_2 emissions and is manifest by the enhanced generation of kaolinite[97,99,100], an increase in continental runoff, extensive dissolution of deep-ocean carbonates, and the characteristic sharp negative C isotope excursion. The period of C release is thought to be less than 20 ka, while the perturbation of C isotope persisted for around 200 ka[97]. Afterwards, the carbon isotope values in marine carbonates slowly recovered to latest Paleocene values.

Unlike the older Lomagundi-age sediments, the assessment of kaolinite content through XRD in the PETM sediments is considerably easier because of the availability of data in the literature. Thus, in this section, we compiled kaolinite content in sediments during the PETM (Supplementary Data 3, Ref. 100 to 114) with the object of quantifying the amount of kaolinite produced during this geological event. Our compilation results show that kaolinite content in the clay-sized sediment fraction increased to over 70% in PETM sediments. By contrast, kaolinite content was lower than 50% and 40% for pre-PETM and post-PETM sediments, respectively. Based on our interpretation of the Lomagundi Event, the increase in kaolinite generation resulting from the increase of temperature and terrestrial runoff could have triggered the kaolinite shuttle from continent to the oceans, leading to the augmentation of nearshore primary productivity. Consequently, sequestration of carbon from the ocean water column and aerial area stored ^{13}C depleted carbon in organic phase, and thus recover the post-PETM $\delta^{13}C_{carb}$ to Paleocene values.

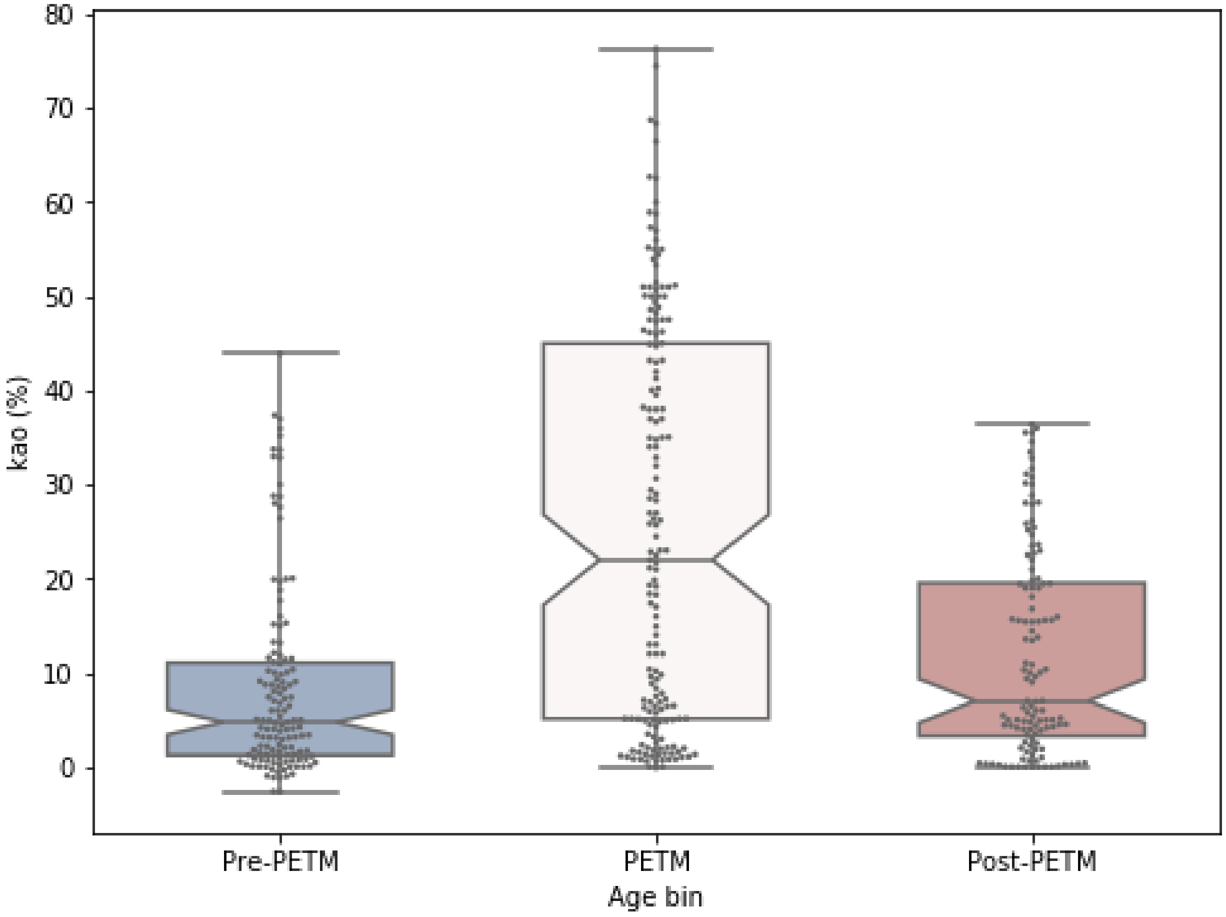

Fig. S11: Kaolinite abundance of sediments during PETM period. Relative kaolinite contents are extracted from literature (see text for extraction method). The data are compiled from 14 references including 17 sampling sections. See Fig. 1 for explanation of figure elements.

References

1 Dorich, R. A., Nelson, D. W. & Sommers, L. E. Algal availability of phosphorus in suspended stream sediments of varying particle-size. *J. Environ. Qual.* **13**, 82-86 (1984). https://doi.org/10.2134/jeq1984.00472425001300010015x

2 Logan, T. J., Oloya, T. O. & Yaksich, S. M. Phosphate characteristics and bioavailability of suspended sediments from streams draining into Lake Erie. *J. Great Lakes Res.* **5**, 112-123 (1979). https://doi.org/10.1016/S0380-1330(79)72136-8

3 He, H. J. et al. Behavior of different phosphorus species in suspended particulate matter in the Changjiang estuary. *Chin. J. Oceanol. Limnol.* **27**, 859-868 (2009). https://doi.org/10.1007/s00343-009-9021-6

4 Hodson, A., Mumford, P. & Lister, D. Suspended sediment and phosphorus in proglacial rivers: bioavailability and potential impacts upon the P status of ice-marginal receiving waters. *Hydrol. Processes* **18**, 2409-2422 (2004).

https://doi.org/10.1002/hyp.1471
5 He, H., Yu, Z., Yao, Q., Chen, H. & Mi, T. The hydrological regime and particulate size control phosphorus form in the suspended solid fraction in the dammed Huanghe (Yellow River). *Hydrobiologia* **638**, 203-211 (2010). 10.1007/s10750-009-0041-1
6 Chase, E. M. & Sayles, F. L. Phosphorus in suspended sediments of the Amazon River. *Estuar. Coast. Shelf Sci.* **11**, 383-391 (1980). https://doi.org/10.1016/S0302-3524(80)80063-6
7 Berner, R. A. & Rao, J. L. Phosphorus in sediments of the Amazon River and estuary - Implications for the global flux of phosphorus to the sea. *Geochim. Cosmochim. Acta* **58**, 2333-2339 (1994). https://doi.org/10.1016/0016-7037(94)90014-0
8 Chakrapani, G. J. & Subramanian, V. Fractionation of heavy metals and phosphorus in suspended sediments of the Yamuna river, India. *Environ. Monit. Assess.* **43**, 117-124 (1996). https://doi.org/10.1007/BF00398602
9 DePinto, J. V., Young, T. C. & Martin, S. C. Algal-available phosphorus in suspended sediments from lower Great Lakes tributaries. *J. Great Lakes Res.* **7**, 311-325 (1981). https://doi.org/10.1016/S0380-1330(81)72059-8
10 Sinclair, P., Beckett, R. & Hart, B. Trace elements in suspended particulate matter from the Yarra River, Australia. *Hydrobiologia* **176**, 239-251 (1989). https://doi.org/10.1007/BF00026559
11 Yao, Q. Z., Du, J. T., Chen, H. T. & Yu, Z. G. Particle-size distribution and phosphorus forms as a function of hydrological forcing in the Yellow River. *Environ. Sci. Pollut. Res.* **23**, 3385-3398 (2016). https://doi.org/10.1007/s11356-015-5567-3
12 Lin, P., Guo, L., Chen, M. & Cai, Y. Distribution, partitioning and mixing behavior of phosphorus species in the Jiulong River estuary. *Mar. Chem.* **157**, 93-105 (2013). https://doi.org/10.1016/j.marchem.2013.09.002
13 Stone, M. & English, M. *Geochemical composition, phosphorus speciation and mass transport of fine-grained sediment in two Lake Erie tributaries*. Proceedings of the Third International Workshop on Phosphorus in Sediments. Springer, pp. 17-29 (1993).
14 Pacini, N. & Gächter, R. Speciation of riverine particulate phosphorus during rain events. *Biogeochem.* **47**, 87-109 (1999). https://doi.org/10.1023/A:1006153302488
15 Kerr, J. G., Burford, M. A., Olley, J. M., Bunn, S. E. & Udy, J. Examining the link between terrestrial and aquatic phosphorus speciation in a subtropical catchment: the role of selective erosion and transport of fine sediments during storm events. *Water Res.* **45**, 3331-3340 (2011). https://doi.org/10.1016/j.watres.2011.03.048
16 Jordan, T. E., Cornwell, J. C., Boynton, W. R. & Anderson, J. T. Changes in phosphorus biogeochemistry along an estuarine salinity gradient: The iron conveyer belt. *Limnol. Oceanogr.* **53**, 172-184 (2008).
17 Dorich, R., Nelson, D. & Sommers, L. Algal availability of sediment phosphorus in drainage water of the Black Creek watershed 1. *J. Environ. Qual.* **9**, 557-563 (1980).

18 Papineau, D. Global biogeochemical changes at both ends of the Proterozoic: Insights from phosphorites. *Astrobiology* **10**, 165–181 (2010). https://doi.org/10.1089/ast.2009.0360
19 Bouton, P. et al. Notice explicative de la Carte géologique de la République du Gabon à 1/200 000, feuille Franceville-Boumango. Eds DGMC – Ministère des Mines, du Pétrole, des Hydrocarbures, Libreville, pp. 79 (2009).
20 Horie, K., Hidaka, H. & Gauthier-Lafaye, F. U-Pb geochronology and geochemistry of zircon from the Franceville series at Bidoudouma, Gabon. *Geochim. Cosmochim. Acta,* **69**, A11 (2005). https://hal.archives-ouvertes.fr/hal-00153287
21 Sawaki, Y. et al. Chronological constraints on the Paleoproterozoic Francevillian Group in Gabon. *Geosci. Front.* **8**, 397-407 (2017). https://doi.org/10.1016/j.gsf.2016.10.001
22 Bros, R., Stille, P., Gauthier-Lafaye, F., Weber, F. & Clauer, N. Sm-Nd isotopic dating of Proterozoic clay material: An example from the Francevillian sedimentary series, Gabon. *Earth Planet. Sci. Lett.* **113**, 207-218 (1992). https://doi.org/10.1016/0012-821X(92)90220-P
23 Ossa, F. O. et al. Two-step deoxygenation at the end of the Paleoproterozoic Lomagundi Event. *Earth Planet. Sci. Lett.* **486**, 70-83 (2018). doi: org/10.1016/j.epsl.2018.01.009
24 Préat, A. et al. Paleoproterozoic high $\delta^{13}C$ dolomites from the Lastoursville and Franceville basins (SE Gabon): Stratigraphic and synsedimentary subsidence implications. *Precambrian Res.* **189**, 212-228 (2011). https://doi.org/10.1016/j.precamres.2011.05.013
25 Canfield, D. E. et al. Oxygen dynamics in the aftermath of the Great Oxidation of Earth's atmosphere. *Proc. Natl. Acad. Sci. USA* **110**, 16736-16741 (2013). https://doi.org/10.1073/pnas.1315570110
26 Kump, L. R. et al. Isotopic evidence for massive oxidation of organic matter following the Great Oxidation Event. *Science* **334**, 1694-1696 (2011). DOI: 10.1126/science.1213999
27 Ossa, F. O. et al. Exceptional preservation of expandable clay minerals in the ca. 2.1 Ga black shales of the Francevillian basin, Gabon and its implication for atmospheric oxygen accumulation. *Chem. Geol.* **362**, 181-192 (2013). doi: 10.1016/j.chemgeo.2013.08.011
28 Bankole, O. M., El Albani, A., Meunier, A., Pambo, F., Paquette, J-L. & Bekker, A. Earth's oldest preserved K-bentonites in the ca. 2.1 Ga Francevillian Basin, Gabon. *Am. J. Sci.* **318**, 409-434 (2018). DOI: https://doi.org/10.2475/04.2018.02
29 Albani, A. et al. Large colonial organisms with coordinated growth in oxygenated environments 2.1 Gyr ago. *Nature* **466**, 100-104 (2010). https://doi.org/10.1038/nature09166
30 Mayika, K. B., Moussavou, M., Prave, A.R., Lepland, A., Mbina, M., & Kirsimäe, K. The Paleoproterozoic Francevillian succession of Gabon and the Lomagundi-Jatuli event. *Geology* **48**, 1099-1104 (2020). doi.org/10.1130/G47651.1

31 Melezhik, V. A., Fallick, A. E., Filippov, M. M. & Larsen, O. Karelian shungite - an indication of 2.0-Ga-old metamorphosed oil-shale and generation of petroleum: geology, lithology and geochemistry. *Earth Sci. Rev.* **47**, 1-40 (1999).
doi: 10.1016/S0012-8252(99)00027-6

32 Geyman, E. C. & Maloof, A. C. A diurnal carbon engine explains ^{13}C-enriched carbonates without increasing the global production of oxygen. *Proc. Natl. Acad. Sci. USA* **116**, 24433-24439 (2019).
doi: org/10.1073/pnas.1908783116

33 Hannah, J. L. et al. *Re-Os geochronology of shungite: A 2.05 Ga fossil oil field in Karelia*. In: Goldschmidt Conference Abstracts. Presented at the 33rd International Geological Congress, Oslo, Norway, p. A351 (2008).

34 Martin, A. P. et al. Multiple Palaeoproterozoic carbon burial episodes and excursions. *Earth Planet. Sci. Lett.* **424**, 226-236 (2015).
https://doi.org/10.1016/j.epsl.2015.05.023

35 Puchtel, I. S. et al. Petrology of mafic lavas within the Onega plateau, central Karelia: evidence for 2.0 Ga plume-related continental crustal growth in the Baltic Shield. *Contrib. Mineral. Petrol.* **130**, 134-153 (1998).
https://doi.org/10.1007/s004100050355

36 Puchtel, I. S., Brügmann, G. E. & Hofmann, A. W. Precise Re–Os mineral isochron and Pb–Nd–Os isotope systematics of a mafic–ultramafic sill in the 2.0 Ga Onega plateau (Baltic Shield). *Earth Planet. Sci. Lett.* **170**, 447-461 (1999).
https://doi.org/10.1016/S0012-821X(99)00118-1

37 Priyatkina, N., Khudoley, A. K., Ustinov, V. N. & Kullerud, K. 1.92 Ga kimberlitic rocks from Kimozero, NW Russia: Their geochemistry, tectonic setting and unusual field occurrence. *Precambrian Res.* **249**, 162-179 (2014).
https://doi.org/10.1016/j.precamres.2014.05.009

38 Stepanova, A., Samsonov, A. & Larionov, A. *The final episode of middle Proterozoic magmatism in the Onega structure: Data on trans-Onega dolerites.* Transactions of the Karelian Research Centre of the Russian Academy of Sciences, Precambrian Geology Series **1**, 3-16 (2014).

39 Asael, D., Rouxel, O., Poulton, S. W., Lyons, T. W. & Bekker, A. Molybdenum record from black shales indicates oscillating atmospheric oxygen levels in the early Paleoproterozoic. *Am. J. Sci.* **318**, 275-299 (2018).
doi: 10.2475/03.2018.01

40 Asael, D. et al. Coupled molybdenum, iron and uranium stable isotopes as oceanic paleoredox proxies during the Paleoproterozoic Shunga Event. *Chem. Geol.* **362**, 193-210 (2013).
https://doi.org/10.1016/j.chemgeo.2013.08.003

41 Scott, C. et al. Pyrite multiple-sulfur isotope evidence for rapid expansion and contraction of the early Paleoproterozoic seawater sulfate reservoir. *Earth Planet. Sci. Lett.* **389**, 95-104 (2014).
https://doi.org/10.1016/j.epsl.2013.12.010

42 Joosu, L. et al. The REE-composition and petrography of apatite in 2 Ga Zaonega Formation, Russia: The environmental setting for phosphogenesis. *Chem. Geol.* **395**, 88-107 (2015).
https://doi.org/10.1016/j.chemgeo.2014.11.013

43 Paiste, K. et al. Multiple sulphur isotope records tracking basinal and global processes in the 1.98 Ga Zaonega Formation, NW Russia. *Chem. Geol.* **499**, 151-164 (2018). https://doi.org/10.1016/j.chemgeo.2018.09.025
44 Paiste, K. et al. The pyrite multiple sulfur isotope record of the 1.98 Ga Zaonega Formation: Evidence for biogeochemical sulfur cycling in a semi-restricted basin. *Earth Planet. Sci. Lett.* **534**, 116092 (2020a). https://doi.org/10.1016/j.epsl.2020.116092
45 Mänd, K. et al. Palaeoproterozoic oxygenated oceans following the Lomagundi–Jatuli Event. *Nat. Geosci.* **13**, 302-306 (2020). https://doi.org/10.1038/s41561-020-0558-5
46 Kipp, M. A., Lepland, A. & Buick, R. Redox fluctuations, trace metal enrichment and phosphogenesis in the ~2.0 Ga Zaonega Formation. *Precambrian Res.* **343**, 105716 (2020). https://doi.org/10.1016/j.precamres.2020.105716
47 Krupenik, V. & Sveshnikova, K. *Correlation of the Onega Parametric Hole with the reference sections of the Onega Structure*. In: The Onega Palaeoproterozoic Structure (Geology, Tectonics, Deep Structure and Minerogeny) (eds. Glushanin L, Sharov N, Shchiptsov V). Institute of Geology, Karelian Research Centre of the Russian Academy of Sciences, pp. 190-195 (2011).
48 Lepland, A. et al. Potential influence of sulphur bacteria on Palaeoproterozoic phosphogenesis. *Nat. Geosci.* **7**, 20-24 (2014). https://doi.org/10.1038/ngeo2005
49 Blättler, C. et al. Two-billion-year-old evaporites capture Earth's great oxidation. *Science* **360**, 320-323 (2018). doi: 10.1126/science.aar2687
50 Paiste, K. et al. Identifying global vs. basinal controls on Paleoproterozoic organic carbon and sulfur isotope records. *Earth Sci. Rev.* **207**, 103230 (2020b). doi.org/10.1016/j.earscirev.2020.103230
51 Kreitsmann, T. et al. Oxygenated conditions in the aftermath of the Lomagundi-Jatuli Event: The carbon isotope and rare earth element signatures of the Paleoproterozoic Zaonega Formation, Russia. *Precambrian Res.* **347**, 105855 (2020). doi.org/10.1016/j.precamres.2020.105855
52 Fedo, C. M., Wayne Nesbitt, H. & Young, G. M. Unraveling the effects of potassium metasomatism in sedimentary rocks and paleosols, with implications for paleoweathering conditions and provenance. *Geology* **23**, 921-924 (1995). doi: org/10.1130/0091-7613(1995)023<0921:UTEOPM>2.3.CO;2
53 Lanson, B., Beaufort, D., Berger, G., Baradat, J. & Lacharpagne, J. C. Illitization of diagenetic kaolinite-to-dickite conversion series; late-stage diagenesis of the Lower Permian Rotliegend Sandstone reservoir, offshore of the Netherlands. *J. Sediment. Res.* **66**, 501-518 (1996). https://doi.org/10.1306/D4268392-2B26-11D7-8648000102C1865D
54 Rye, R. & Holland, H. D. Paleosols and the evolution of atmospheric oxygen: a critical review. *Am. J. Sci.* **298**, 621-672 (1998). doi: 10.2475/ajs.298.8.621
55 Nedachi, Y., Nedachi, M., Bennett, G. & Ohmoto, H. Geochemistry and mineralogy of the 2.45 Ga Pronto paleosols, Ontario, Canada. *Chem. Geol.* **214**, 21-44 (2005). doi: org/10.1016/j.chemgeo.2004.08.026

56 Teitler, Y. et al. Ubiquitous occurrence of basaltic-derived paleosols in the Late Archean Fortescue Group, Western Australia. *Precambrian Res.* **267**, 1-27 (2015). doi: org/10.1016/j.precamres.2015.05.014
57 Marmo, J. S. *The lower Proterozoic Hokkalampi paleosol in north Karelia, eastern Finland*. Springer, p:41-66 (1992).
58 de Wall, H., Pandit, M. K. & Chauhan, N. K. Paleosol occurrences along the Archean–Proterozoic contact in the Aravalli craton, NW India. *Precambrian Res.* **216**, 120-131 (2012). doi: org/10.1016/j.precamres.2012.06.017
59 Nesbitt, H. W. & Young, G. Early Proterozoic climates and plate motions inferred from major element chemistry of lutites. *Nature* **299**, 715 (1982). doi: org/10.1038/299715a0
60 Maynard, J. B. Chemistry of modern soils as a guide to interpreting Precambrian paleosols. *J. Geol.* **100**, 279-289 (1992). https://doi.org/10.1086/629632
61 Rye, R., & Holland, H. D. Geology and geochemistry of Paleosols developed on the Hekpoort Basalt, Pretoria Group, South Africa. *Am. J. Sci.* **300**, 85-141 (2000). https://doi.org/10.2475/ajs.300.2.85
62 Stafford, S. L. Precambrian paleosols as indicators of paleoenvironments on the early Earth. University of Pittsburgh (2007).
63 Soomer, S. et al. High-CO2, acidic and oxygen-starved weathering at the Fennoscandian Shield at the Archean-Proterozoic transition. *Precambrian Res.* **327**, 68-80 (2019). doi: org/10.1016/j.precamres.2019.03.001
64 Babechuk, M. G. et al. Pervasively anoxic surface conditions at the onset of the Great Oxidation Event: New multi-proxy constraints from the Cooper Lake paleosol. *Precambrian Res.* **323**, 126-163 (2019). https://doi.org/10.1016/j.precamres.2018.12.029
65 Macfarlane, A. W., Danielson, A. & Holland, H. D. Geology and major and trace element chemistry of late Archean weathering profiles in the Fortescue Group, Western Australia: implications for atmospheric pO_2. *Precambrian Res.* **65**, 297-317 (1994). https://doi.org/10.1016/0301-9268(94)90110-4
66 Yang, W., Holland, H. D. & Rye, R. Evidence for low or no oxygen in the late Archean atmosphere from the ~2.76 Ga Mt. Roe #2 paleosol, Western Australia: Part 3. *Geochim. Cosmochim. Acta* **66**, 3707-3718 (2002). https://doi.org/10.1016/S0016-7037(01)00673-1
67 Babechuk, M. G. & Kamber, B. S. The Flin Flon paleosol revisited. *Can. J. Earth Sci.* **50**, 1223-1243 (2013). https://doi.org/10.1139/cjes-2013-0076
68 Medaris, L. G., Driese, S. G. & Stinchcomb, G. E. The Paleoproterozoic Baraboo paleosol revisited: Quantifying mass fluxes of weathering and metasomatism, chemical climofunctions, and atmospheric pCO_2 in a chemically heterogeneous protolith. *Precambrian Res.* **301**, 179-194 (2017). https://doi.org/10.1016/j.precamres.2017.06.010
69 Murakami, T., Matsuura, K. & Kanzaki, Y. Behaviors of trace elements in Neoarchean and Paleoproterozoic paleosols: Implications for atmospheric oxygen evolution and continental oxidative weathering. *Geochim. Cosmochim. Acta* **192**, 203-219 (2016).

https://doi.org/10.1016/j.gca.2016.07.008
70 Toma, J., Holmden, C., Shakotko, P., Pan, Y. & Ootes, L. Cr isotopic insights into ca. 1.9 Ga oxidative weathering of the continents using the Beaverlodge Lake paleosol, Northwest Territories, Canada. *Geobiology* **17**, 467-489 (2019). https://doi.org/10.1111/gbi.12342
71 Panahi, A., Young, G. M. & Rainbird, R. H. Behavior of major and trace elements (including REE) during Paleoproterozoic pedogenesis and diagenetic alteration of an Archean granite near Ville Marie, Québec, Canada. *Geochim. Cosmochim. Acta* **64**, 2199–2220 (2000). doi.org/10.1016/S0016-7037(99)00420-2
72 Bachan, A. & Kump, L. R. The rise of oxygen and siderite oxidation during the Lomagundi Event. *Proc. Natl. Acad. Sci. USA* **112**, 6562-6567 (2015). doi: org/10.1073/pnas.1422319112
73 Smith, E. A., Mayfield, C. I. & Wong, P. T. S. Physical and chemical characterization of selected natural apatites in synthetic and natural aqueous solutions. *Water Air Soil Pollut.* **8**, 401–415 (1977). https://doi.org/10.1007/BF00228655
74 McLennan, S. M., Hemming, S., McDaniel, D. K. & Hanson, G. N. *Geochemical approaches to sedimentation, provenance, and tectonic*s. In: Geological Society of America Special Papers. Geological Society of America, pp. 21-40 (1993). https://doi.org/10.1130/SPE284-p21
75 Liivamägi, S. et al. Petrology, mineralogy and geochemical climofunctions of the Neoproterozoic Baltic paleosol. *Precambrian Res.* **256**, 170-188 (2015). https://doi.org/10.1016/j.precamres.2014.11.008
76 Medaris, L. G., Boerboom, T. J., Jicha, B. R. & Singer, B. S. Metasaprolite in the McGrath Gneiss, Minnesota, USA: Viewing Paleoproterozoic weathering through a veil of metamorphism and metasomatism. *Precambrian Res.* **257**, 83-93 (2015). https://doi.org/10.1016/j.precamres.2014.11.027
77 Haile, N. S. Calculation of paleolatitudes from paleomagnetic poles. *Geology* **3**, 174 (1975). https://doi.org/10.1130/0091-7613(1975)3<174:COPFPP>2.0.CO;2
78 Buchan, K. L., Mortensen, J. K., Card, K. D. & Percival, J. A. Paleomagnetism and U-Pb geochronology of diabase dyke swarms of Minto block, Superior Province, Quebec, Canada. *Can. J. Earth Sci.* **35**, 1054-1069 (1998). https://doi.org/10.1139/e98-054
79 Halls, H. C. & Heaman, L. M. The paleomagnetic significance of new U-Pb age data from the Molson dyke swarm, Cauchon Lake area, Manitoba. *Can. J. Earth Sci.* **37**, 957-966 (2000). https://doi.org/10.1139/e00-010
80 Halls, H. C., Davis, D. & Stott, G. M. *Paleomagnetism, geochronology and geochemistry of several Proterozoic mafic dike swarms in northwestern Ontario.* Ontario Geological Survey (2005).
81 Irving, E., Baker, J., Hamilton, M. & Wynne, P. J. Early Proterozoic geomagnetic field in western Laurentia: Implications for paleolatitudes, local rotations and stratigraphy. *Precambrian Res.* **129**, 251-270 (2004). https://doi.org/10.1016/j.precamres.2003.10.002

82 Mertanen, S., Vuollo, J. I., Huhma, H., Arestova, N. A. & Kovalenko, A. Early Paleoproterozoic–Archean dykes and gneisses in Russian Karelia of the Fennoscandian Shield—New paleomagnetic, isotope age and geochemical investigations. *Precambrian Res.* **144**, 239-260 (2006). https://doi.org/10.1016/j.precamres.2005.11.005

83 Strik, G., de Wit, M. J. & Langereis, C. G. Palaeomagnetism of the Neoarchaean Pongola and Ventersdorp Supergroups and an appraisal of the 3.0–1.9Ga apparent polar wander path of the Kaapvaal Craton, Southern Africa. *Precambrian Res.* **153**, 96-115 (2007). https://doi.org/10.1016/j.precamres.2006.11.006

84 Strik, G., Blake, T. S., Zegers, T. E., White, S. H. & Langereis, C. G. Palaeomagnetism of flood basalts in the Pilbara Craton, Western Australia: Late Archaean continental drift and the oldest known reversal of the geomagnetic field. *J. Geophys. Res. B: Solid Earth* **108**, B12 (2003). https://doi.org/10.1029/2003JB002475

85 Lubnina, N. V. & Slabunov, A. I. The Karelian Craton in the structure of the Kenorland Supercontinent in the Neoarchean: New paleomagnetic and isotope geochronology data on granulites of the Onega complex. Moscow Univ. *Geol. Bull.* **72**, 377-390 (2017). https://doi.org/10.3103/S0145875217060072

86 McKinney, W., *Data Structures for statistical computing in python*. In: van der Walt, S., Millman, J. (Eds.). Proceedings of the 9th Python in Science Conference. pp. 56-61 (2010). https://doi.org/10.25080/Majora-92bf1922-00a

87 Harper, M. et al. *Python-ternary: Ternary plots in python.* Version 1.0.6. Zenodo (2015). https://doi.org/10.5281/zenodo.2628066

88 Hunter, J. D. Matplotlib: A 2D graphics environment. *Computing in Science Engineering* **9**, 90–95 (2007). https://doi.org/10.1109/MCSE.2007.55

89 Waskom, M. et al. *Seaborn: statistical data visualization*. Version 0.10.1. Zenodo (2020). https://doi.org/10.5281/zenodo.3767070

90 Buchan, K. L., Mertanen, S., Park, R. G., Pesonen, L. J., Elming, S. Å., Abrahamsen, N., & Bylund, G. Comparing the drift of Laurentia and Baltica in the Proterozoic: the importance of key palaeomagnetic poles. *Tectonophysics* **319**, 167–198 (2000).

91 Land, J. S., Tsikos, H., Cousins, D., Luvizotto, G., & Zack, T. Origin of red beds and paleosols in the Palaeoproterozoic Transvaal and Olifansthoek Supergroups of South Africa: provenance versus metasomatic controls. *Geol. J.* **53**, 191–202 (2018).

92 Yang, X., Zhang, Y., Bao, S., Shen, C. Separation and recovery of vanadium from a sulfuric-acid leaching solution of stone coal by solvent extraction using trialkylamine. *Sep. Purif. Technol.* **164**, 49-55 (2016).

93 Hao, W., Flynn, S. L., Alessi, D. S. & Konhauser, K. O. Change of the point of zero net proton charge (pH_{PZNPC}) of clay minerals with ionic strength. *Chem. Geol.* **493**, 458-467 (2018). doi: org/10.1016/j.chemgeo.2018.06.023

94 Hao, W. et al. The impact of ionic strength on the proton reactivity of clay minerals. *Chem. Geol.* **529**, 119294 (2019). doi: org/10.1016/j.chemgeo.2019.119294

95 Liu, Y. et al. Acid-base properties of kaolinite, montmorillonite and illite at marine ionic strength. *Chem. Geol.* **483**, 191-200 (2018).

96 Westall, J. C. *FITEQL: A Computer Program for Determination of Chemical Equilibrium Constants from Experimental Data*. Department of Chemistry, Oregon State University (1982).
97 McInerney, F. A. & Wing, S. L. The Paleocene-Eocene Thermal Maximum: A perturbation of carbon cycle, climate, and biosphere with implications for the future. *Annu. Rev. Earth Planet Sci.* **39**, 489-516 (2011).
98 Kennett, J. P. & Stott L. D. Abrupt deep-sea warming, palaeoceanographic changes and benthic extinctions at the end of the Palaeocene. *Nature* **353**, 225–29 (1991).
99 Zachos, J. C., et al. Rapid acidification of the ocean during the Paleocene-Eocene Thermal Maximum. *Science* **308**, 1611–15 (2005).
100 Chen, Z., Ding, Z., Yang, S., Zhang, C. & Wang, X. Increased precipitation and weathering across the Paleocene-Eocene Thermal Maximum in central China. *Geochem. Geophys. Geosyst.* **17**, 2286-2297 (2016).
101 Cramer, B. S., Aubry, M. P., Miller, K. G., Olsson, R. K., Wright, J. D. & Kent, D. V. An exceptional chronologic, isotopic, and clay mineralogic record of the latest Paleocene thermal maximum, Bass River, NJ, ODP 174AX. *Bull. Soc. Geol. Fr.* **170**, 883-897 (1999).
102 Gibson, T. G., Bybell, L. M. & Mason, D. B. Stratigraphic and climatic implications of clay mineral changes around the Paleocene/Eocene boundary of the northeastern US margin. *Sediment. Geol.* **134**, 65-92 (2000).
103 Gibson, T. G., Bybell, L. M. & Owens, J. P. Latest Paleocene lithologic and biotic events in neritic deposits of southwestern New Jersey. *Paleoceanogr.* **8**, 495-514 (1993).
104 Handley, L., et al. Changes in the hydrological cycle in tropical East Africa during the Paleocene–Eocene Thermal Maximum. *Palaeogeogr. Palaeoclimatol. Palaeoecol.* **329**, 10-21 (2012).
105 Harding, I. C., et al. Sea-level and salinity fluctuations during the Paleocene–Eocene thermal maximum in Arctic Spitsbergen. *Earth Planet. Sci. Lett.* **303**, 97-107 (2011).
106 John, C. M., Banerjee, N. R., Longstaffe, F. J., Sica, C., Law, K. R. & Zachos, J. C. Clay assemblage and oxygen isotopic constraints on the weathering response to the Paleocene-Eocene thermal maximum, east coast of North America. *Geol.* **40**, 591-594 (2012).
107 Kelly, D. C., Zachos, J. C., Bralower, T. J. & Schellenberg, S. A. Enhanced terrestrial weathering/runoff and surface ocean carbonate production during the recovery stages of the Paleocene-Eocene thermal maximum. *Paleoceanogr.* **20**, (2005).
108 Kemp, S. J., Ellis, M. A., Mounteney, I. & Kender, S. Palaeoclimatic implications of high-resolution clay mineral assemblages preceding and across the onset of the Palaeocene–Eocene Thermal Maximum, North Sea Basin. *Clay Miner.* **51**, 793-813 (2016).
109 Pujalte, V., Baceta, J. I. & Schmitz, B. A massive input of coarse-grained siliciclastics in the Pyrenean Basin during the PETM: the missing ingredient in a coeval abrupt change in hydrological regime. *Clim. Past* **11**, 1653-1672 (2015).
110 Robert, C. & Chamley, H. Development of early Eocene warm climates, as inferred from clay mineral variations in oceanic sediments. *Palaeogeogr. Palaeoclimatol. Palaeoecol.* **89**, 315-331 (1991).
111 Robert, C. & Kennett, J. P. Paleocene and Eocene kaolinite distribution in the South Atlantic and Southern Ocean: Antarctic climatic and paleoceanographic implications. *Mar. Geol.* **103**, 99-110 (1992).

112 Schulte, P., Scheibner, C. & Speijer, R. P. Fluvial discharge and sea-level changes controlling black shale deposition during the Paleocene–Eocene Thermal Maximum in the Dababiya Quarry section, Egypt. *Chem. Geol.* **285**, 167-183 (2011).

113 Tateo, F. Clay Minerals at the Paleocene–Eocene Thermal Maximum: Interpretations, Limits, and Perspectives. *Miner.* **10**, 1073 (2020).

114 Wieczorek, R., Fantle, M. S., Kump, L. R. & Ravizza, G. Geochemical evidence for volcanic activity prior to and enhanced terrestrial weathering during the Paleocene Eocene Thermal Maximum. *Geochim. Cosmochim. Acta* **119**, 391-410 (2013).
