# Supplementary material for: The kaolinite shuttle links the Great Oxidation and Lomagundi events
Source: Nat Commun. 2021 May 19;12:2944. doi: 10.1038/s41467-021-23304-8 (PMC8134571; doi:10.1038/s41467-021-23304-8)
Supplement: Supplementary file 2 — Description of Additional Supplementary Files [file 41467_2021_23304_MOESM2_ESM.pdf]

Description of Additional Supplementary Files

File Name: Supplementary Data 1

Description: Paleosol geochemical data across the GOE

File Name: Supplementary Data 2

Description: Paleosol paleogeography across the GOE

File Name: Supplementary Data 3

Description: Kaolinite data compilation across the PETM
